# Mendelian randomisation analysis of circulating adipokines and C‐reactive protein on breast cancer risk

**DOI:** 10.1002/ijc.32947

**Published:** 2020-03-13

**Authors:** Timothy Robinson, Richard M. Martin, James Yarmolinsky

**Affiliations:** ^1^ Population Health Sciences, Bristol Medical School University of Bristol Bristol UK; ^2^ MRC Integrative Epidemiology Unit, Population Health Sciences, Bristol Medical School University of Bristol Bristol UK; ^3^ University Hospitals Bristol, NHS Foundation Trust, National Institute for Health Research Bristol Biomedical Research Centre University of Bristol Bristol UK

**Keywords:** adipokines, C‐reactive protein, breast cancer, Mendelian randomisation

## Abstract

Circulating adipokines and C‐reactive protein (CRP) have been linked to breast cancer risk in observational epidemiological studies. The causal nature of these associations is unclear because of the susceptibility of conventional observational designs to residual confounding, reverse causation and other forms of bias. Mendelian randomisation (MR) uses genetic variants as proxies for risk factors to strengthen causal inference in observational settings. We performed a MR analysis to evaluate the causal relevance of six previously reported circulating adipokines [adiponectin, hepatocyte growth factor (HGF), interleukin‐6, leptin receptor, plasminogen activator inhibitor‐1 and resistin] and CRP in risk of overall and oestrogen receptor‐stratified breast cancer in up to 122,977 cases and 105,974 controls of European ancestry. Genetic instruments were constructed from single‐nucleotide polymorphisms robustly (*p* < 5 × 10^−8^) associated with risk factors in genome‐wide association studies. Colocalisation was performed as a sensitivity analysis to examine whether findings reflected shared causal variants or genomic confounding. In MR analyses, there was evidence for an association of HGF with oestrogen receptor‐negative cancer (odds ratio per standard deviation increase: 1.17, 95% confidence interval: 1.01–1.35; *p* = 0.035) but little evidence for associations of other adipokines or CRP with overall or oestrogen receptor‐stratified breast cancer. Colocalisation analysis suggested that the association of HGF with oestrogen receptor‐negative breast cancer was unlikely to reflect a causal association. Collectively, these findings do not support an important aetiological role of various adipokines or CRP in overall or oestrogen receptor‐specific breast cancer risk.

## Abstract 2

**What's new?**

Adipokines and C‐reactive protein have been linked to breast cancer risk in observational studies. But while these molecular mediators potentially connect excess adiposity with breast cancer, whether the associations are causal in nature is uncertain. Here, a Mendelian randomization approach was employed to estimate the causal effect of six circulating adipokines and C‐reactive protein on breast cancer risk. Overall, among 122,977 cases and 105,974 controls, analyses provided little evidence supporting a causal role for these molecular markers in breast cancer etiology. The findings warrant investigation of other mediators that could explain apparent links between adiposity and increased breast cancer risk.

**Abbreviations**
BCAC: Breast Cancer Association Consortium
CRP: C‐reactive protein
ER−: oestrogen receptor‐negative
ER+: oestrogen receptor‐positive
GWAS: genome‐wide association study
HGF: hepatocyte growth factor
IL‐6: interleukin‐6
InSIDE: Instrument Strength Independent of Direct Effect
IV: instrumental variable
IVW: inverse‐variance weighted
MB: megabase
MR: Mendelian randomisation
OR: odds ratio
PAI‐1: plasminogen activator inhibitor‐1
SD: standard deviation
SNP: single‐nucleotide polymorphism

## Introduction

Elevated body mass index is an important modifiable risk factor for breast cancer.^1^ With the discovery of adipose tissue as a functioning endocrine organ, much attention has focused on a putative role of adipokines – cytokines and hormones released by adipose tissue – as molecular mediators linking excess adiposity to breast cancer.^2^, ^3^, ^4^ Two adipokines in particular – leptin and adiponectin – have been strongly implicated in breast cancer aetiology. *In vitro* studies indicate that leptin may promote human breast cancer cell proliferation and adiponectin may exert antiproliferative effects^5^ and meta‐analyses of observational studies support their opposing roles in breast cancer risk.^4^, ^6^, ^7^ Likewise, several other adipokines including hepatocyte growth factor (HGF), interleukin‐6 (IL‐6), plasminogen activator inhibitor‐1 (PAI‐1) and resistin have been linked to breast cancer risk in observational studies, albeit less consistently.^8^, ^9^, ^10^ Prediagnostic C‐reactive protein (CRP), a systemic marker of inflammation that is synthesised in part by adipose tissue,^11^ has also been associated with breast cancer risk in prospective observational studies.^12^ Collectively, these observational findings suggest that pharmacological targeting of adipokines or CRP could be an effective strategy for breast cancer prevention among overweight or obese women. However, the causal nature of these risk factors in breast cancer risk, and thus their suitability as intervention targets, is unclear. This is because of the uncertain relevance of *in vitro* studies to humans, and the susceptibility of conventional observational analyses to residual confounding and reverse causation, all of which undermines causal inference.^13^, ^14^

Mendelian randomisation (MR) uses genetic variants as instruments (‘proxies’) for risk factors (including those that are potentially modifiable) to generate more reliable evidence on the causal effects of these factors on disease outcomes and so inform potential intervention strategies.^15^, ^16^ The use of genetic variants as instruments minimises confounding and precludes reverse causation as germline genotype is largely independent of lifestyle and environmental factors and is fixed at conception. The statistical power and precision of MR analysis can be increased by employing a ‘two‐sample MR’ framework in which summary genetic association data from independent samples representing genetic variant–exposure and genetic variant–outcome associations are synthesised in order to estimate causal effects.^17^

More formally, MR is a form of instrumental variable (IV) analysis that can generate unbiased causal estimates of effects of risk factors on disease and health‐related outcomes if the following conditions are met: (*i*) the instrument is robustly associated with the exposure of interest, (*ii*) there is no confounding of the instrument–outcome relationship and (*iii*) there is no pathway through which an instrument influences an outcome except through the exposure (‘exclusion restriction criterion’).

Given uncertainty surrounding the role of various previously reported adipokines and CRP in breast cancer aetiology, we performed two‐sample MR analyses to evaluate the causal relevance of circulating adiponectin, HGF, IL‐6, leptin receptor, PAI‐1, resistin and CRP in overall and oestrogen receptor‐stratified breast cancer risk.

## Materials and Methods

### Study population

Summary genome‐wide association study (GWAS) statistics were obtained from genome‐wide meta‐analyses on 122,977 breast cancer cases and 105,974 controls of European ancestry.^18^ Summary statistics were also obtained from analyses on 69,501 oestrogen receptor‐positive (ER+) and 21,468 oestrogen receptor‐negative (ER−) breast cancer cases (105,974 controls) of European ancestry.^18^ Genotype data for a subset of the overall sample (61,282 cases and 45,494 controls) were obtained by direct genotyping using an Illumina Custom Infinium array (OncoArray) consisting of approximately 530,000 single‐nucleotide polymorphisms (SNPs) or by imputation with reference to the 10,000 Genomes Project Phase 3 reference panel.^19^ All SNPs with a call rate of < 95%, evidence of violation of Hardy–Weinberg equilibrium (*p* < 1.0 × 10^−7^ in controls or *p* < 1.0 × 10^−12^ in cases) and SNPs with concordance of <98% among 5,280 duplicate sample pairs were removed. An additional 1,128 SNPs where the cluster plot was judged to be inadequate were removed. In imputation, the following SNPs were additionally removed: those with a minor allele frequency of <1%, a call rate of <98%, and those that could not be linked to the 1,000 Genomes Project or differed significantly in frequency from this panel. In total, 469,364 of the 533,631 SNPs that were manufactured on the OncoArray were used in imputation. Results for OncoArray analyses were combined with those from the previous Illumina iSelect Collaborative Oncological Gene‐Environment Study (iCOGS) genotyping project (46,785 cases; 42,892 controls) along with 11 other breast cancer GWAS (14,910 cases; 17,588 controls), using a fixed‐effects meta‐analysis. The OncoArray, iCOGS and individual GWAS were adjusted for principal components of ancestry and OncoArray and iCOGS analyses were further adjusted for country and study, respectively. All participating studies had the approval of their appropriate ethics review board and all participants provided informed consent.

### Instrument construction

Genetic instruments to proxy risk factors were constructed either using a ‘monogenic’ approach (restricted to *cis*‐acting variants, i.e. located ≤1 MB of the transcription start site of the protein‐coding gene) or a ‘polygenic’ approach (combining *cis*‐ and *trans*‐acting variants, independent of genomic position), depending on the number of genome‐wide significant (*p* < 5 × 10^−8^) variants available to proxy each respective risk factor. When few SNPs are available as instruments, the restriction of an instrument to *cis*‐variants can help to minimise horizontal pleiotropy (an instrument influencing an outcome through one or more biological pathways independent to that of the exposure), a violation of the exclusion restriction criterion, as *cis*‐variants are more likely to have direct effects on protein levels than *trans*‐variants (i.e. those >1 MB of the transcription start site of the protein‐coding gene).^16^ Genetic instruments to proxy HGF, IL‐6, leptin receptor and resistin were constructed by obtaining *cis*‐acting SNPs robustly associated with these markers (*p* < 5 × 10^−8^) in GWAS of individuals of European ancestry that were replicated in independent samples. For risk factors with ≥3 independent (*r* ^2^ < 0.01) *cis*‐ or *trans*‐SNPs available as proxies in GWAS of individuals of European ancestry (adiponectin, CRP, PAI‐1), these SNPs were combined into multi‐allelic instruments to increase the variance in the risk factor explained by the instrument.^20^, ^21^, ^22^ As sensitivity analyses for adiponectin, CRP and PAI‐1, effect estimates generated from multi‐allelic instruments were compared to those obtained from instruments consisting of weakly correlated (*r* ^2^ < 0.15) *cis‐*variants to investigate horizontal pleiotropy in primary multi‐allelic models.^23^ Across GWAS used to instrument various traits, sample sizes varied from 3,301 to 133,449 participants, mean ages of participants varied from 43.7 to 59.0 years, the proportion of females in the sample varied from 48.9 to 50.0%, and all participants were of European ancestry. Study‐level information on sample size, mean age and percentage of female participants are presented in Table 1. Estimates of data set overlap across adipokine or CRP and breast cancer analyses are presented in Supporting Information Table S1. Complete summary genetic association data for all SNPs used to instrument each trait is presented in Supporting Information Table S2.

**Table 1:** Characteristics of studies used to construct instrumental variables

Trait	Author	Sample size	Mean age	% Female
Adiponectin	Dastani *et al*.^20^	30,708–38,276	NA	NA
CRP	Ligthart *et al*.^22^	204,402	NA	NA
HGF	Sun *et al*.^24^	3,301	43.7	48.9
IL‐6	Swerdlow *et al*.^25^	133,449	59.0	49.0
Leptin receptor	Sun *et al*.^24^	3,301	43.7	48.9
PAI‐1	Huang *et al*.^21^	30,395	54.3	50.0
Resistin	Sun *et al*.^24^	3,301	43.7	48.9

Abbreviations: CRP, C‐reactive protein; HGF, hepatocyte growth factor; IL‐6, interleukin‐6; NA, not available in publication; PAI‐1, plasminogen activator inhibitor‐1.

### Statistical analysis

*R* ^2^ and *F*‐statistics were calculated to assess the strength of instruments and to examine for weak instrument bias (i.e. reduced statistical power to reject the null hypothesis when an instrument explains a limited proportion of variance in an exposure), using previously reported methods.^26^ For instruments constructed using individual *cis*‐variants (HGF, leptin receptor, resistin and sensitivity analyses for PAI‐1), effect estimates were generated using the Wald ratio and standard errors were approximated using the delta method. For instruments constructed using ≥3 independent variants (adiponectin, CRP, PAI‐1), effect estimates were generated using inverse‐variance weighted (IVW) random‐effects models to account for overdispersion in models.^27^ For instruments constructed using multiple weakly correlated *cis*‐variants (IL‐6 and sensitivity analyses for adiponectin, CRP), effect estimates were generated using IVW random‐effects models with adjustment for correlations between variants.^28^ If underdispersion was present in random‐effects models, the residual standard error was set to 1. For instruments constructed using multiple independent variants (adiponectin, CRP, PAI‐1), MR‐Egger (regression and intercept parameter) and weighted median estimation were used to evaluate for violations of the exclusion restriction criterion.^27^, ^29^ MR‐Egger regression can provide unbiased estimates of causal effects even when all IVs in an instrument are invalid provided that the instrument strength independent of direct effect (InSIDE) assumption is met (i.e. that there is no association between the strength of IV–exposure associations and the magnitude of horizontal pleiotropy). The MR‐Egger intercept can provide a formal statistical test for directional pleiotropy (i.e. where the net horizontal pleiotropic effect across an instrument does not average to zero). The weighted median estimate can provide unbiased estimates of causal effects when at least 50% of the information in an instrument derives from valid IVs. This approach has two advantages over MR‐Egger in that it provides improved precision as compared to the latter and does not rely on the InSIDE assumption. For adiponectin, CRP and PAI‐1 analyses, we also visually examined for evidence of potential outliers which may be indicative of horizontal pleiotropy by generating scatter plots, forest plots and funnel plots. Leave‐one‐out permutation analysis was also performed for these three traits to examine whether any results were driven by an influential SNP within instruments.

As an additional sensitivity analysis, colocalisation was performed to examine whether two traits showing evidence of an association in MR analyses share the same causal variant at a given locus. This is important to perform for analyses in which an instrument is restricted to either a single variant or a single gene region as findings may be more susceptible to bias through genetic confounding (i.e., exposure and outcomes are influenced by distinct causal variants that are in linkage disequilibrium with each other). The coloc R package uses approximate Bayes factor computation to generates posterior probabilities that associations between two traits represent each of the following configurations: (*i*) neither trait has a genetic association in the region (*H*_0_), (*ii*) only the first trait has a genetic association in the region (*H*_1_), (*iii*) only the second trait has a genetic association in the region (*H*_2_), (*iv*) both traits are associated but have different causal variants (*H*_3_) and (*v*) both traits are associated and share a single causal variant (*H*_4_).^30^ An assumption of coloc is that there is at most one causal variant per trait within the genomic region examined. Colocalisation analysis was performed for MR analyses showing evidence of association (*p* < 0.05) by generating windows ±100 kb from the top SNP used to instrument the exposure. As a convention, a posterior probability of ≥0.80 was used to indicate support for a configuration tested. To evaluate assumptions of the coloc package (i.e. there is at most one causal variant per trait) we also generated regional Manhattan plots for SNP‐risk factor and SNP–breast cancer associations to visually inspect whether there was evidence of multiple independent causal variants within the region examined.

To account for multiple testing across each breast cancer endpoint (overall breast cancer risk, ER+ breast cancer risk, ER− breast cancer risk), a Bonferroni correction was used to establish a *p*‐value threshold of <0.007 (false‐positive rate = 0.05/7 risk factors). All statistical analyses were performed using R version 3.3.1.

### Data availability

Summary genetic association data for all traits examined in this study were obtained from previously published analyses and can be made available upon reasonable request.^18^, ^20^, ^21^, ^22^, ^23^, ^24^, ^25^

## Results

For each risk factor, the number of SNPs included in the instrument and estimates of instrument strength (*R* ^2^ and *F*‐statistics) are presented in Table 2. Across risk factors assessed, *F*‐statistics ranged from 40.1 to 3,872.7, suggesting that analyses were unlikely to suffer from weak instrument bias.^31^ Conservative estimates of sample overlap across GWAS data sets used ranged from 0.0 to 7.3% (Supporting Information Table S1). Given minimal sample overlap and likely absence of weak instrument bias, the presence of overlap across some GWAS data sets is unlikely to have introduced bias into these analyses.

**Table 2:** Number of SNPs included in instrument, estimate of the proportion of variance in risk factor explained by the instrument (*R* ^2^) and *F*‐statistic for each instrument, across all adipokines and CRP

Risk factor	Number of SNPs in instrument	*R* ^2^	*F*‐statistic
Adiponectin	8	0.016	60.8
CRP	45	0.035	119.4
HGF	1	0.012	40.1
IL‐6	3	0.002	51.4
Leptin receptor	1	0.54	3,872.7
PAI‐1	3	0.0064	65.5
Resistin	1	0.030	103.3

Abbreviations: CRP, C‐reactive protein; HGF, hepatocyte growth factor; IL‐6, interleukin‐6; PAI‐1, plasminogen activator inhibitor‐1; SNPs, single‐nucleotide polymorphisms.

*R* ^2^ indicates proportion of variance in risk factor explained by genetic instrument.

**Figure 1:**
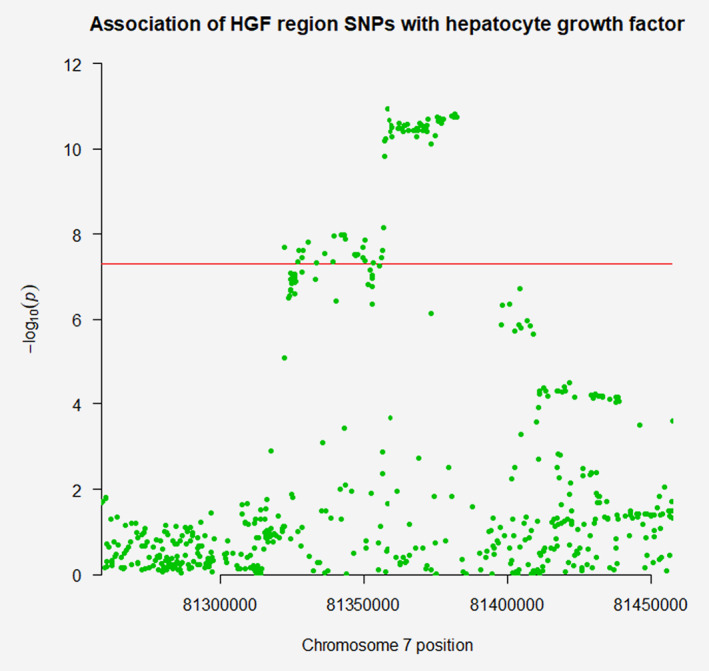
Regional Manhattan plot of associations of single‐nucleotide polymorphisms (SNPs) with circulating hepatocyte growth factor (HGF) ±100 kb from the SNP used to proxy HGF (rs5745695) in the *HGF* region. [Color figure can be viewed at wileyonlinelibrary.com]

**Figure 2:**
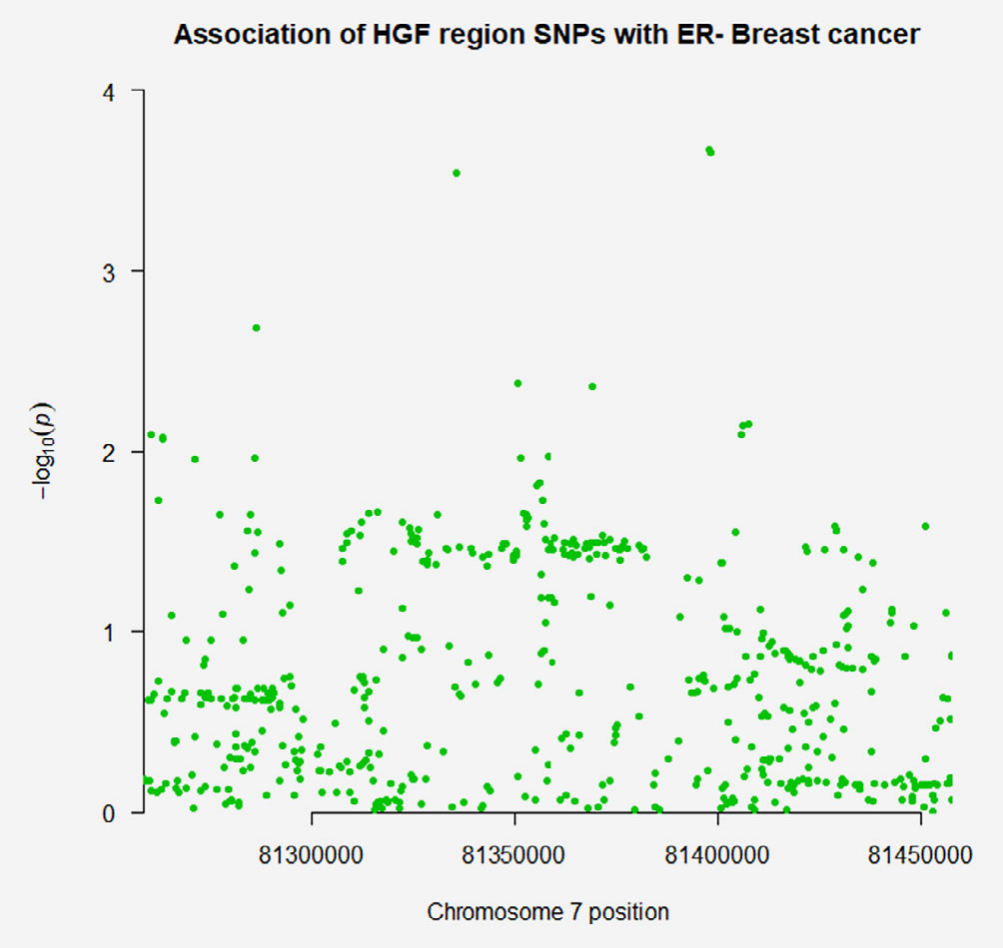
Regional Manhattan plot of associations of single‐nucleotide polymorphisms (SNPs) with oestrogen receptor‐negative breast cancer ±100 kb from the SNP used to proxy hepatocyte growth factor (rs5745695) in the *HGF* region. [Color figure can be viewed at wileyonlinelibrary.com]

In MR analyses, there was little evidence to suggest associations of any of the adipokines or CRP with overall breast cancer risk (Table 3). In ER status‐stratified analyses, there was evidence for an association of HGF with ER− breast cancer risk [odds ratio (OR) per standard deviation (SD) increase: 1.17, 95% confidence interval (CI): 1.01–1.35; *p* = 0.035]. This finding did not reach statistical significance using a Bonferroni‐corrected threshold (*p* < 0.007). In sensitivity analyses, there was little evidence that this association colocalised (posterior probability *H*_4_ = 0.055). Complete colocalisation analysis results are presented in Supporting Information Table S3. Regional Manhattan plots examining the association of all SNPs ±100 kb from the SNP used to instrument HGF for their association with this adipokine (Fig. 1) and with ER− breast cancer (Fig. 2) did not appear to support the presence of one or more independent causal variants for SNP‐ER− breast cancer analyses.

**Table 3:** Effect estimates per unit increase in adipokines or CRP on overall and oestrogen receptor‐stratified breast cancer risk

Risk factor	Overall breast cancer		ER+ breast cancer		ER− breast cancer	
	OR (95% CI)	*p*‐Value	OR (95% CI)	*p*‐Value	OR (95% CI)	*p*‐Value
Adiponectin	1.06 (0.81–1.40)	0.66	0.98 (0.71–1.35)	0.91	1.19 (0.92–1.54)	0.18
CRP	1.03 (0.94–1.13)	0.48	1.04 (0.95–1.14)	0.40	1.05 (0.93–1.19)	0.41
HGF	1.01 (0.93–1.10)	0.77	1.01 (0.92–1.11)	0.86	1.17 (1.01–1.35)	0.035
IL‐6	1.09 (0.96–1.25)	0.18	1.12 (0.96–1.31)	0.14	1.00 (0.79–1.27)	0.99
Leptin receptor	1.00 (0.99–1.01)	0.63	1.00 (0.99–1.01)	0.81	1.00 (0.98–1.02)	0.78
PAI‐1	1.03 (0.80–1.33)	0.83	0.98 (0.78–1.24)	0.87	1.05 (0.65–1.68)	0.85
Resistin	0.98 (0.91–1.04)	0.48	0.98 (0.91–1.06)	0.61	0.99 (0.87–1.11)	0.81

Abbreviations: CI, confidence interval; CRP, C‐reactive protein; ER+, oestrogen receptor positive; ER−, oestrogen receptor negative; HGF, hepatocyte growth factor; IL‐6, interleukin‐6; OR, odds ratio; PAI‐1, plasminogen activator inhibitor‐1.

Effect estimates represent the effect of a one unit increase in: natural log‐transformed adiponectin, CRP, IL‐6 and PAI‐1 and standardised HGF, leptin receptor and resistin.

In all other ER‐stratified analyses, there was little evidence of association of other adipokines or CRP with breast cancer outcomes.

Findings for adiponectin, PAI‐1 and CRP in sensitivity analyses using *cis*‐SNP instruments for both overall and oestrogen receptor‐stratified breast cancer were consistent with those from the primary analysis (Supporting Information Table S4). Findings were also consistent for these three risk factors when MR‐Egger and weighted median estimation was employed to test for evidence of exclusion restriction criterion violations (Supporting Information Table S5). There were no clear violations of MR assumptions in forest, funnel and scatter plots generated (Supporting Information Figs. S1–S36).

## Discussion

Our MR analyses in up to 122,977 breast cancer cases and 105,974 controls do not support an important aetiological role of six adipokines or CRP in breast cancer risk. In analyses stratified on oestrogen receptor status, there was some evidence for an association of HGF with risk of oestrogen receptor‐negative breast cancer. However, this finding did not achieve statistical significance based on a Bonferroni‐corrected threshold to account for multiple comparisons. Additionally, sensitivity analysis testing the probability that both traits share the same causal variant found little evidence for colocalisation.

Our findings are not consistent with some previous conventional observational analyses, notably those examining the association of adiponectin and leptin with breast cancer.^6^, ^7^, ^8^, ^9^, ^12^ A meta‐analysis of 15 cohort and case–control studies reported a 34% risk reduction (95% CI: 13–50%) when comparing ‘highest’ to ‘lowest’ adiponectin levels, with moderate heterogeneity across studies (*I* ^2^ = 53%).^6^ Likewise, a meta‐analysis of 35 case–control studies reported a standardised mean difference in serum leptin levels of 0.46 ng/ml (95% CI:  0.31–0.60, *I* ^2^  =  93.5%) when comparing breast cancer cases to controls.^7^ However, four prospective studies examining the relationship between prediagnostic leptin and breast cancer risk have shown conflicting results: two have supported positive associations (upper *vs*. lower quartile: OR 1.94, 95% CI: 1.37–2.75; upper *vs* lower tertile: OR 1.98, 95% CI: 1.20–3.29),^32^, ^33^ whereas two found little evidence of association (upper *vs* lower quartile: OR 1.39, 95% CI: 0.93–2.09; upper *vs*. lower tertile: OR 0.83, 95% CI: 0.51–1.37).^3^, ^34^ Divergent findings across studies could be attributable to residual confounding (due to unmeasured or imprecisely measured confounders) or reverse causation in some conventional observational analyses. For example, the ability to disentangle highly correlated measured adipokine levels from each other and from other metabolic perturbations associated with the obese phenotype (e.g. insulin resistance, chronic inflammation) may be limited when using conventional multivariable regression methods.^35^ Additionally, it is possible that previously reported associations of inflammatory markers like CRP with breast cancer could represent the effect of early stage or latent breast cancer on subsequent inflammation levels.^36^

Strengths of this analysis include the use of a MR approach to appraise the relationship of adipokines and CRP with breast cancer risk which should be less prone to confounding than conventional observational analyses and cannot be influenced by reverse causation. The use of a two‐sample summary data MR framework afforded these analyses increased statistical power and precision by exploiting summary genetic data from several large genome‐wide association studies. The incorporation of colocalisation analysis permitted evaluation of whether associations present were driven through genetic confounding or shared common causal variants. Finally, through restricting summary genetic association data to that obtained in individuals of European descent and adjusted for principal components of ancestry, these analyses reduced the possibility of confounding through population substructures.

There are several limitations to these analyses. First, since analyses were performed using summary genetic data in aggregate, this precluded stratification according to menopausal status. Around 85% of samples included within the Breast Cancer Association Consortium (BCAC) data used were classified as postmenopausal at diagnosis, suggesting that findings were unlikely to be biased markedly by the presence of premenopausal or perimenopausal participants.^18^ Second, though attempts were made to circumvent potential violations of MR assumptions in our analyses through the use of *cis*‐acting variants as primary instruments and in sensitivity analyses, we cannot rule out the possibility that false negative findings may have arisen through horizontally pleiotropic pathways biasing our findings toward the null. An assumption of the colocalisation package employed is that there is at most one causal variant per trait within the region examined. If both traits have a genetic association in the region examined, a low posterior probability generated for *H*_4_ (probability that both traits share a single causal variant) could reflect the presence of multiple shared causal variants within that region. However, posterior probabilities generated in this analysis provided evidence that only HGF was likely to have an association in the *HGF* region examined, suggesting that this assumption was unlikely to be violated. Fourth, it is not known whether circulating levels of adipokines represent a good surrogate for tissue‐specific levels in breast or adipose tissue. Fifth, we were unable to examine potential nonlinear associations of adipokines or CRP with breast cancer risk.

Beyond adipokines and inflammatory pathways, there are several other molecular mechanisms through which excess adiposity may influence subsequent risk of breast cancer.^37^ For example, recent MR evidence in the BCAC (*N* = 98, 842 cases; 83, 464 controls) has suggested that elevated fasting insulin, a consequence of higher adiposity, increases breast cancer risk (OR per SD increase = 1.71, 95% CI: 1.26–2.31).^38^ Along with the recognised role of oestrogen levels in development of ER+ breast cancer, the hyperactivation of insulin‐like growth factor pathways and oxidative stress have also been implicated as molecular mediators in breast carcinogenesis, both of which may potentially influence disease through dysregulation of mammalian target of rapamycin pathway signalling.^37^ Further examination of the putative role of these molecular pathways in carcinogenesis could help to identify potential pharmacological targets for the chemoprevention of breast cancer in high‐risk groups and, ultimately, help to reduce the burden of breast cancer.

In conclusion, our MR analyses suggest that several adipokines and CRP are unlikely to causally influence breast cancer risk. Further exploration of potential molecular mediators linking adiposity to elevated breast cancer risk remains warranted.

## Conflict of Interest

T.R. has received educational funding from Daiichi‐Sankyo and Amgen. These funding sources have not influenced the authorship or content of this manuscript.

## Supporting information


**Appendix** **S1**: Supporting information tables and figures
**Table S1.** Estimates of sample overlap across exposure and BCAC datasets
**Table S2.** Summary genetic association data used across each adipokine and C‐reactive protein
**Table S3.** Posterior probabilities under differing hypotheses relating the associations between hepatocyte growth factor and oestrogen receptor‐negative breast cancer risk
**Table S4.** Effect estimates per unit increase in adiponectin, C‐reactive protein, and plasminogen activator inhibitor‐1 on overall and oestrogen receptor stratified breast cancer risk using conservative (cis‐SNP) instruments
**Table S5.** Sensitivity analyses for adiponectin, C‐reactive protein, and plasminogen activator inhibitor‐1 using MR‐Egger and weighted median estimates
**Figure S1.** Scatterplot of associations of individual SNPs used to proxy adiponectin with adiponectin and overall breast cancer risk along with slopes obtain from the following models: inverse‐variance weighted, MR‐Egger, weighted median
**Figure S2.** Scatterplot of associations of individual SNPs used to proxy adiponectin with adiponectin and oestrogen receptor‐positive breast cancer risk along with slopes obtain from the following models: inverse‐variance weighted, MR‐Egger, weighted median
**Figure S3.** Scatterplot of associations of individual SNPs used to proxy adiponectin with adiponectin and oestrogen receptor‐negative breast cancer risk along with slopes obtain from the following models: inverse‐variance weighted, MR‐Egger, weighted median
**Figure S4.** Leave‐one‐out analysis iteratively removing one SNP from the instrument used to proxy adiponectin and re‐calculating the association of adiponectin with overall breast cancer risk
**Figure S5.** Leave‐one‐out analysis iteratively removing one SNP from the instrument used to proxy adiponectin and re‐calculating the association of adiponectin with oestrogen receptor‐positive breast cancer risk
**Figure S6.** Leave‐one‐out analysis iteratively removing one SNP from the instrument used to proxy adiponectin and re‐calculating the association of adiponectin with oestrogen receptor‐negative breast cancer risk
**Figure S7.** Forest plot presenting individual SNP estimates and multi‐allelic instrument estimates for the association of adiponectin with overall breast cancer risk
**Figure S8.** Forest plot presenting individual SNP estimates and multi‐allelic instrument estimates for the association of adiponectin with oestrogen receptor‐positive breast cancer risk
**Figure S9.** Forest plot presenting individual SNP estimates and multi‐allelic instrument estimates for the association of adiponectin with oestrogen receptor‐negative breast cancer risk
**Figure S10.** Funnel plot presenting the effect estimate and inverse of the standard error for the causal estimate of each single‐nucleotide polymorphism examining the association of adiponectin with overall breast cancer risk along with slopes obtained from inverse‐variance weighted and MR‐Egger models
**Figure S11.** Funnel plot presenting the effect estimate and inverse of the standard error for the causal estimate of each single‐nucleotide polymorphism examining the association of adiponectin with oestrogen receptor‐positive breast cancer risk along with slopes obtained from inverse‐variance weighted and MR‐Egger models
**Figure S12.** Funnel plot presenting the effect estimate and inverse of the standard error for the causal estimate of each single‐nucleotide polymorphism examining the association of adiponectin with oestrogen receptor‐negative breast cancer risk along with slopes obtained from inverse‐variance weighted and MR‐Egger models
**Figure S13.** Scatterplot of associations of individual SNPs used to proxy Creactive protein with C‐reactive protein and overall breast cancer risk along with slopes obtain from the following models: inverse‐variance weighted, MR‐Egger, weighted median
**Figure S14.** Scatterplot of associations of individual SNPs used to proxy Creactive protein with C‐reactive protein and oestrogen receptor‐positive breast cancer risk along with slopes obtain from the following models: inverse‐variance weighted, MR‐Egger, weighted median
**Figure S15.** Scatterplot of associations of individual SNPs used to proxy Creactive protein with C‐reactive protein and oestrogen receptor‐negative breast cancer risk along with slopes obtain from the following models: inverse‐variance weighted, MR‐Egger, weighted median
**Figure S16.** Leave‐one‐out analysis iteratively removing one SNP from the instrument used to proxy C‐reactive protein and re‐calculating the association of C‐reactive protein with overall breast cancer risk
**Figure S17.** Leave‐one‐out analysis iteratively removing one SNP from the instrument used to proxy C‐reactive protein and re‐calculating the association of C‐reactive protein with oestrogen receptor‐positive breast cancer risk
**Figure S18.** Leave‐one‐out analysis iteratively removing one SNP from the instrument used to proxy C‐reactive protein and re‐calculating the association of C‐reactive protein with oestrogen receptor‐negative breast cancer risk
**Figure S19.** Forest plot presenting individual SNP estimates and multi‐allelic instrument estimates for the association of C‐reactive protein with overall breast cancer risk
**Figure S20.** Forest plot presenting individual SNP estimates and multi‐allelic instrument estimates for the association of C‐reactive protein with oestrogen receptor‐positive breast cancer risk
**Figure S21.** Forest plot presenting individual SNP estimates and multi‐allelic instrument estimates for the association of C‐reactive protein with oestrogen receptornegative breast cancer risk
**Figure S22.** Funnel plot presenting the effect estimate and inverse of the standard error for the causal estimate of each single‐nucleotide polymorphism examining the association of C‐reactive protein with overall breast cancer risk along with slopes obtained from inverse‐variance weighted and MR‐Egger models
**Figure S23.** Funnel plot presenting the effect estimate and inverse of the standard error for the causal estimate of each single‐nucleotide polymorphism examining the association of C‐reactive protein with oestrogen receptor‐positive breast cancer risk along with slopes obtained from inverse‐variance weighted and MR‐Egger models
**Figure S24.** Funnel plot presenting the effect estimate and inverse of the standard error for the causal estimate of each single‐nucleotide polymorphism examining the association of C‐reactive protein with oestrogen receptor‐negative breast cancer risk along with slopes obtained from inverse‐variance weighted and MR‐Egger models
**Figure S25.** Scatterplot of associations of individual SNPs used to proxy plasminogen activator inhibitor‐1 with plasminogen activator inhibitor‐1 and overall breast cancer risk along with slopes obtain from the following models: inverse‐variance weighted, MR‐Egger, weighted median
**Figure S26.** Scatterplot of associations of individual SNPs used to proxy plasminogen activator inhibitor‐1 with plasminogen activator inhibitor‐1 and oestrogen receptor‐positive breast cancer risk along with slopes obtain from the following models: inverse‐variance weighted, MR‐Egger, weighted median
**Figure S27.** Scatterplot of associations of individual SNPs used to proxy plasminogen activator inhibitor‐1 with plasminogen activator inhibitor‐1 and oestrogen receptor‐negative breast cancer risk along with slopes obtain from the following models: inverse‐variance weighted, MR‐Egger, weighted median
**Figure S28.** Leave‐one‐out analysis iteratively removing one SNP from the instrument used to proxy plasminogen activator inhibitor‐1 and re‐calculating the association of plasminogen activator inhibitor‐1 with overall breast cancer risk
**Figure S29.** Leave‐one‐out analysis iteratively removing one SNP from the instrument used to proxy plasminogen activator inhibitor‐1 and re‐calculating the association of plasminogen activator inhibitor‐1 with oestrogen receptor‐positive breast cancer risk
**Figure S30.** Leave‐one‐out analysis iteratively removing one SNP from the instrument used to proxy plasminogen activator inhibitor‐1 and re‐calculating the association of plasminogen activator inhibitor‐1 with oestrogen receptor‐negative breast cancer risk
**Figure S31.** Forest plot presenting individual SNP estimates and multi‐allelic instrument estimates for the association of plasminogen activator inhibitor‐1 with overall breast cancer risk
**Figure S32.** Forest plot presenting individual SNP estimates and multi‐allelic instrument estimates for the association of plasminogen activator inhibitor‐1 with oestrogen receptor‐positive breast cancer risk
**Figure S33.** Forest plot presenting individual SNP estimates and multi‐allelic instrument estimates for the association of plasminogen activator inhibitor‐1 with oestrogen receptor‐negative breast cancer risk
**Figure S34.** Funnel plot presenting the effect estimate and inverse of the standard error for the causal estimate of each single‐nucleotide polymorphism examining the association of plasminogen activator inhibitor‐1 with overall breast cancer risk along with slopes obtained from inverse‐variance weighted and MR‐Egger models
**Figure S35.** Funnel plot presenting the effect estimate and inverse of the standard error for the causal estimate of each single‐nucleotide polymorphism examining the association of plasminogen activator inhibitor‐1 with oestrogen receptor‐positive breast cancer risk along with slopes obtained from inverse‐variance weighted and MR‐Egger models
**Figure S36.** Funnel plot presenting the effect estimate and inverse of the standard error for the causal estimate of each single‐nucleotide polymorphism examining the association of plasminogen activator inhibitor‐1 with oestrogen receptor‐negative breast cancer risk along with slopes obtained from inverse‐variance weighted and MR‐Egger models
