# Supplementary material for: Mendelian randomisation analysis of circulating adipokines and C‐reactive protein on breast cancer risk
Source: Int J Cancer. 2020 Mar 13;147(6):1597–603. doi: 10.1002/ijc.32947 (PMC7497166; doi:10.1002/ijc.32947)
Supplement: Supplementary file 1 — Appendix S1: Supporting information tables and figures Table S1. Estimates of sample overlap across exposure and BCAC datasets Table S2. Summary genetic association data used across each adipokine and C‐reactive protein Table S3. Posterior probabilities under differing hypotheses relating the associations between hepatocyte growth factor and oestrogen receptor‐negative breast cancer risk Table S4. Effect estimates per unit increase in adiponectin, C‐reactive protein, and plasminogen activator inhibitor‐1 on overall and oestrogen receptor stratified breast cancer risk using conservative (cis‐SNP) instruments Table S5. Sensitivity analyses for adiponectin, C‐reactive protein, and plasminogen activator inhibitor‐1 using MR‐Egger and weighted median estimates Figure S1. Scatterplot of associations of individual SNPs used to proxy adiponectin with adiponectin and overall breast cancer risk along with slopes obtain from the following models: inverse‐variance weighted, MR‐Egger, weighted median Figure S2. Scatterplot of associations of individual SNPs used to proxy adiponectin with adiponectin and oestrogen receptor‐positive breast cancer risk along with slopes obtain from the following models: inverse‐variance weighted, MR‐Egger, weighted median Figure S3. Scatterplot of associations of individual SNPs used to proxy adiponectin with adiponectin and oestrogen receptor‐negative breast cancer risk along with slopes obtain from the following models: inverse‐variance weighted, MR‐Egger, weighted median Figure S4. Leave‐one‐out analysis iteratively removing one SNP from the instrument used to proxy adiponectin and re‐calculating the association of adiponectin with overall breast cancer risk Figure S5. Leave‐one‐out analysis iteratively removing one SNP from the instrument used to proxy adiponectin and re‐calculating the association of adiponectin with oestrogen receptor‐positive breast cancer risk Figure S6. Leave‐one‐out analysis iteratively removing one SNP from the instrumen [file IJC-147-1597-s001.pdf]

Mendelian Randomization Analysis of Circulating Adipokines and C-reactive Protein on Breast Cancer Risk

Timothy Robinson, Richard M Martin, James Yarmolinsky

Supplementary Table 1. Estimates of sample overlap across exposure and BCAC datasets

Supplementary Table 2. Summary genetic association data used across each adipokine and C-reactive protein

Supplementary Table 3. Posterior probabilities under differing hypotheses relating the associations between hepatocyte growth factor and oestrogen receptor-negative breast cancer risk

Supplementary Table 4. Effect estimates per unit increase in adiponectin, C-reactive protein, and plasminogen activator inhibitor-1 on overall and oestrogen receptor stratified breast cancer risk using conservative (cis-SNP) instruments

Supplementary Table 5. Sensitivity analyses for adiponectin, C-reactive protein, and plasminogen activator inhibitor-1 using MR-Egger and weighted median estimates

Supplementary Figure 1. Scatterplot of associations of individual SNPs used to proxy adiponectin with adiponectin and overall breast cancer risk along with slopes obtain from the following models: inverse-variance weighted, MR-Egger, weighted median

Supplementary Figure 2. Scatterplot of associations of individual SNPs used to proxy adiponectin with adiponectin and oestrogen receptor-positive breast cancer risk along with slopes obtain from the following models: inverse-variance weighted, MR-Egger, weighted median

Supplementary Figure 3. Scatterplot of associations of individual SNPs used to proxy adiponectin with adiponectin and oestrogen receptor-negative breast cancer risk along with slopes obtain from the following models: inverse-variance weighted, MR-Egger, weighted median

Supplementary Figure 4. Leave-one-out analysis iteratively removing one SNP from the instrument used to proxy adiponectin and re-calculating the association of adiponectin with overall breast cancer risk

Supplementary Figure 5. Leave-one-out analysis iteratively removing one SNP from the instrument used to proxy adiponectin and re-calculating the association of adiponectin with oestrogen receptor-positive breast cancer risk

Supplementary Figure 6. Leave-one-out analysis iteratively removing one SNP from the instrument used to proxy adiponectin and re-calculating the association of adiponectin with oestrogen receptor-negative breast cancer risk

Supplementary Figure 7. Forest plot presenting individual SNP estimates and multi-allelic instrument estimates for the association of adiponectin with overall breast cancer risk

Supplementary Figure 8. Forest plot presenting individual SNP estimates and multi-allelic instrument estimates for the association of adiponectin with oestrogen receptor-positive breast cancer risk

Supplementary Figure 9. Forest plot presenting individual SNP estimates and multi-allelic instrument estimates for the association of adiponectin with oestrogen receptor-negative breast cancer risk

Supplementary Figure 10. Funnel plot presenting the effect estimate and inverse of the standard error for the causal estimate of each single-nucleotide polymorphism examining the association of adiponectin with overall breast cancer risk along with slopes obtained from inverse-variance weighted and MR-Egger models

Supplementary Figure 11. Funnel plot presenting the effect estimate and inverse of the standard error for the causal estimate of each single-nucleotide polymorphism examining the association of adiponectin with oestrogen receptor-positive breast cancer risk along with slopes obtained from inverse-variance weighted and MR-Egger models

Supplementary Figure 12. Funnel plot presenting the effect estimate and inverse of the standard error for the causal estimate of each single-nucleotide polymorphism examining the association of adiponectin with oestrogen receptor-negative breast cancer risk along with slopes obtained from inverse-variance weighted and MR-Egger models

Supplementary Figure 13. Scatterplot of associations of individual SNPs used to proxy C-reactive protein with C-reactive protein and overall breast cancer risk along with slopes obtain from the following models: inverse-variance weighted, MR-Egger, weighted median

Supplementary Figure 14. Scatterplot of associations of individual SNPs used to proxy C-reactive protein with C-reactive protein and oestrogen receptor-positive breast cancer risk along with slopes obtain from the following models: inverse-variance weighted, MR-Egger, weighted median

Supplementary Figure 15. Scatterplot of associations of individual SNPs used to proxy C-reactive protein with C-reactive protein and oestrogen receptor-negative breast cancer risk along with slopes obtain from the following models: inverse-variance weighted, MR-Egger, weighted median

Supplementary Figure 16. Leave-one-out analysis iteratively removing one SNP from the instrument used to proxy C-reactive protein and re-calculating the association of C-reactive protein with overall breast cancer risk

Supplementary Figure 17. Leave-one-out analysis iteratively removing one SNP from the instrument used to proxy C-reactive protein and re-calculating the association of C-reactive protein with oestrogen receptor-positive breast cancer risk

Supplementary Figure 18. Leave-one-out analysis iteratively removing one SNP from the instrument used to proxy C-reactive protein and re-calculating the association of C-reactive protein with oestrogen receptor-negative breast cancer risk

Supplementary Figure 19. Forest plot presenting individual SNP estimates and multi-allelic instrument estimates for the association of C-reactive protein with overall breast cancer risk

Supplementary Figure 20. Forest plot presenting individual SNP estimates and multi-allelic instrument estimates for the association of C-reactive protein with oestrogen receptor-positive breast cancer risk

Supplementary Figure 21. Forest plot presenting individual SNP estimates and multi-allelic instrument estimates for the association of C-reactive protein with oestrogen receptor-negative breast cancer risk

Supplementary Figure 22. Funnel plot presenting the effect estimate and inverse of the standard error for the causal estimate of each single-nucleotide polymorphism examining the association of C-reactive protein with overall breast cancer risk along with slopes obtained from inverse-variance weighted and MR-Egger models

Supplementary Figure 23. Funnel plot presenting the effect estimate and inverse of the standard error for the causal estimate of each single-nucleotide polymorphism examining the association of C-reactive protein with oestrogen receptor-positive breast cancer risk along with slopes obtained from inverse-variance weighted and MR-Egger models

Supplementary Figure 24. Funnel plot presenting the effect estimate and inverse of the standard error for the causal estimate of each single-nucleotide polymorphism examining the association of C-reactive protein with oestrogen receptor-negative breast cancer risk along with slopes obtained from inverse-variance weighted and MR-Egger models

Supplementary Figure 25. Scatterplot of associations of individual SNPs used to proxy plasminogen activator inhibitor-1 with plasminogen activator inhibitor-1 and overall breast cancer risk along with slopes obtain from the following models: inverse-variance weighted, MR-Egger, weighted median

Supplementary Figure 26. Scatterplot of associations of individual SNPs used to proxy plasminogen activator inhibitor-1 with plasminogen activator inhibitor-1 and oestrogen receptor-positive breast cancer risk along with slopes obtain from the following models: inverse-variance weighted, MR-Egger, weighted median

Supplementary Figure 27. Scatterplot of associations of individual SNPs used to proxy plasminogen activator inhibitor-1 with plasminogen activator inhibitor-1 and oestrogen receptor-negative breast cancer risk along with slopes obtain from the following models: inverse-variance weighted, MR-Egger, weighted median

Supplementary Figure 28. Leave-one-out analysis iteratively removing one SNP from the instrument used to proxy plasminogen activator inhibitor-1 and re-calculating the association of plasminogen activator inhibitor-1 with overall breast cancer risk

Supplementary Figure 29. Leave-one-out analysis iteratively removing one SNP from the instrument used to proxy plasminogen activator inhibitor-1 and re-calculating the association of plasminogen activator inhibitor-1 with oestrogen receptor-positive breast cancer risk

Supplementary Figure 30. Leave-one-out analysis iteratively removing one SNP from the instrument used to proxy plasminogen activator inhibitor-1 and re-calculating the association of plasminogen activator inhibitor-1 with oestrogen receptor-negative breast cancer risk

Supplementary Figure 31. Forest plot presenting individual SNP estimates and multi-allelic instrument estimates for the association of plasminogen activator inhibitor-1 with overall breast cancer risk

Supplementary Figure 32. Forest plot presenting individual SNP estimates and multi-allelic instrument estimates for the association of plasminogen activator inhibitor-1 with oestrogen receptor-positive breast cancer risk

Supplementary Figure 33. Forest plot presenting individual SNP estimates and multi-allelic instrument estimates for the association of plasminogen activator inhibitor-1 with oestrogen receptor-negative breast cancer risk

Supplementary Figure 34. Funnel plot presenting the effect estimate and inverse of the standard error for the causal estimate of each single-nucleotide polymorphism examining the association of plasminogen activator inhibitor-1 with overall breast cancer risk along with slopes obtained from inverse-variance weighted and MR-Egger models

Supplementary Figure 35. Funnel plot presenting the effect estimate and inverse of the standard error for the causal estimate of each single-nucleotide polymorphism examining the association of plasminogen activator inhibitor-1 with oestrogen receptor-positive breast cancer risk along with slopes obtained from inverse-variance weighted and MR-Egger models

Supplementary Figure 36. Funnel plot presenting the effect estimate and inverse of the standard error for the causal estimate of each single-nucleotide polymorphism examining the association of plasminogen activator inhibitor-1 with oestrogen receptor-negative breast cancer risk along with slopes obtained from inverse-variance weighted and MR-Egger models

Supplementary Table 1. Estimates of sample overlap across exposure and BCAC datasets

Instrument	Overlapping Cohort	Sample size after QC in BCAC	As a percentage of total BCAC population (228,951)
Adiponectin	NHS	3,392	1.5
C-reactive protein	EPIC	7,057	3.1
	WHI	9,521	4.2
Interleukin-6	EPIC	7,057	3.1
	WHI	9,521	4.2

QC= Quality control, BCAC= Breast Cancer Association Consortium, NHS = Nurses Health Study, EPIC = European Prospective Investigation into Cancer and Nutrition, WHI = Women’s Health Initiative

Supplementary Table 2. Summary genetic association data used across each adipokine and C-reactive protein

Trait	rsid	Effect allele	Non-effect allele	Effect allele frequency	Beta	SE
Adiponectin	rs3001032	T	C	0.70	-0.02	0.004
	rs1108842	C	A	0.50	0.03	0.004
	rs6810075	T	C	0.60	0.06	0.004
	rs998584	C	A	0.50	0.03	0.005
	rs2980879	T	A	0.70	0.03	0.005
	rs7955516	C	A	0.40	0.02	0.004
	rs601339	G	A	0.20	0.03	0.005
	rs2925979	T	C	0.30	-0.04	0.005
	rs12922394	T	C	0.10	-0.08	0.01
	rs731839	G	A	0.35	-0.03	0.004
C-reactive Protein	rs1051338	G	T	0.31	0.024	0.004
	rs10832027	G	A	0.33	-0.026	0.004
	rs10838687	G	T	0.22	-0.031	0.004
	rs112635299	T	G	0.02	-0.107	0.017
	rs12202641	T	C	0.39	-0.023	0.004
	rs12960928	C	T	0.27	0.024	0.004
	rs12995480	T	C	0.17	-0.031	0.005
	rs1441169	G	A	0.53	-0.025	0.004
	rs1490384	T	C	0.51	-0.025	0.004
	rs1514895	A	G	0.71	-0.027	0.004
	rs1558902	A	T	0.41	0.034	0.004
	rs1582763	A	G	0.37	-0.022	0.004
	rs17658229	C	T	0.05	0.056	0.010
	rs178810	T	C	0.56	0.020	0.004
	rs1880241	G	A	0.48	-0.028	0.004
	rs2239222	G	A	0.36	0.035	0.004
	rs2315008	T	G	0.31	-0.023	0.004
	rs2352975	C	T	0.3	0.025	0.004
	rs2710804	C	T	0.37	0.021	0.004
	rs2836878	G	A	0.27	0.043	0.004
	rs2891677	C	T	0.46	-0.020	0.004
	rs4092465	A	G	0.35	-0.027	0.004
	rs4246598	A	C	0.46	0.022	0.004
	rs469772	T	C	0.19	-0.031	0.005
	rs4774590	A	G	0.35	-0.022	0.004
	rs6001193	G	A	0.35	-0.028	0.004
	rs7121935	A	G	0.38	-0.022	0.004
	rs75460349	A	C	0.97	0.086	0.014
	rs9271608	G	A	0.22	0.042	0.005
	rs9284725	C	A	0.24	0.027	0.004
	rs9385532	T	C	0.33	-0.026	0.004
	rs2293476	C	G	0.23	0.030	0.004
	rs1805096	G	A	0.39	0.104	0.004
	rs4129267	C	T	0.39	0.088	0.004
	rs2794520	C	T	0.33	0.182	0.004
	rs10925027	T	C	0.4	0.036	0.004
	rs1260326	T	C	0.39	0.073	0.004
	rs13409371	A	G	0.43	0.048	0.004

	rs13233571	C	T	0.12	0.057	0.005
	rs4841132	G	A	0.09	0.065	0.006
	rs340005	A	G	0.38	0.03	0.004
	rs10521222	C	T	0.05	0.104	0.011
	rs2852151	A	G	0.40	0.025	0.004
	rs4420638	A	G	0.18	0.229	0.006
	rs1800961	C	T	0.03	0.112	0.011
HGF	rs5745695	A	G	0.76	0.19	0.03
Interleukin 6	rs7529229	T	C	0.55	0.086	0.012
	rs4845371	T	C	0.43	0.062	0.013
	rs12740969	T	G	0.49	0.078	0.013
Leptin receptor	rs3790438	A	T	0.18	-1.37	0.02
PAI-I	rs2227631	A	G	0.59	0.073	0.007
	rs6486122	T	C	0.69	0.046	0.007
	rs6976053	T	C	0.48	0.048	0.007
Resistin	rs34124816	C	A	0.04	-0.61	0.06

SE= Standard error, HGF= Hepatocyte growth factor, PAI-1= Plasminogen activator inhibitor-1. Effect estimates and standard errors are scaled as follows: natural log-transformed adiponectin, C-reactive protein, interleukin-6, and plasminogen activator inhibitor-1 and standardised hepatocyte growth factor, leptin receptor, and resistin.

Supplementary Table 3. Posterior probabilities under differing hypotheses relating the associations between hepatocyte growth factor and oestrogen receptor-negative breast cancer risk

Configuration	H_0	H_1	H_2	H_3	H_4
	7.25×10^{-7}	0.93	1.47×10^{-8}	1.90×10^{-2}	5.50×10^{-2}

H_0 = neither hepatocyte growth factor nor oestrogen receptor-negative breast cancer has a genetic association in the region, H_1 = only hepatocyte growth factor has a genetic association in the region, H_2 = only oestrogen receptor-negative breast cancer has a genetic association in the region, H_3 = both hepatocyte growth factor and oestrogen receptor-negative breast cancer are associated but have different causal variants, H_4= both hepatocyte growth factor and oestrogen receptor-negative breast cancer are associated and share a single causal variant

Supplementary Table 4. Effect estimates per unit increase in adiponectin, C-reactive protein, and plasminogen activator inhibitor-1 on overall and oestrogen receptor stratified breast cancer risk using conservative (*cis*-SNP) instruments*

**Risk factor**	**Overall breast cancer**	**ER+ breast cancer**	**ER- breast cancer**
	**OR (95% CI)**	**OR (95% CI)**	**OR (95% CI)**
Adiponectin	0.97 (0.92-1.02)	0.98 (0.92-1.05)	0.98 (0.89-1.09)
C-reactive protein	1.01 (0.95-1.07)	1.02 (0.96-1.10)	1.04 (0.94-1.16)
PAI-1	1.10 (0.93-1.30)	1.16 (0.94-1.42)	1.13 (0.83-1.53)

SNP = single-nucleotide polymorphism, OR = Odds Ratio, 95% CI = 95% Confidence Interval, PAI-1 = Plasminogen activator inhibitor-1.

**Cis*-SNP instruments were constructed as follows: adiponectin was instrumented using four variants within *ADIPOQ* (rs6810075, rs16861209, rs17366568, rs3774261), C-reactive protein was instrumented using four variants within *CRP* (rs3093077, rs1205, rs1130864, rs1800947), and plasminogen activator inhibitor-1 was instrumented using one variant within *SERPINE* (rs2227631).

Effect estimates represent the effect of a one unit increase in: natural log-transformed adiponectin, C-reactive protein, and plasminogen activator inhibitor-1.

Supplementary Table 5. Sensitivity analyses for adiponectin, C-reactive protein, and plasminogen activator inhibitor-1 using MR-Egger and weighted median estimates

**Trait**	**Model**	**Overall Breast cancer OR (95% CI); *P***	**ER+ Breast cancer OR (95% CI); *P***	**ER- Breast cancer OR (95% CI); *P***
Adiponectin	MR-Egger regression	1.04 (0.91-1.20); 0.53	1.07 (0.93-1.23); 0.33	1.03 (0.86-1.23); 0.77
	MR-Egger intercept parameter	1.00 (0.99-1.01); 0.83	1.00 (0.99-1.01); 0.58	1.00 (0.99-1.01); 0.72
	Weighted median estimate	1.03 (0.97-1.09); 0.35	1.06 (1.00-1.14); 0.06	1.07 (0.97-1.18); 0.16
C-reactive protein	MR-Egger regression	1.24 (0.55-2.78); 0.62	1.27 (0.50-3.24); 0.63	0.95 (0.45-1.99); 0.89
	MR-Egger intercept parameter	0.99 (0.96-1.02); 0.70	0.99 (0.95-1.03); 0.58	1.01 (0.98-1.04); 0.54
	Weighted median estimate	1.08 (0.91-1.29); 0.37	1.02 (0.84-1.25); 0.84	0.98 (0.72-1.34); 0.92
PAI-1	MR-Egger regression	0.58 (0.17-2.02); 0.55	0.54 (0.22-1.32); 0.40	0.50 (0.03-7.71); 0.71
	MR-Egger intercept parameter	1.03 (0.96-1.11); 0.53	1.04 (0.98-1.09); 0.41	1.04 (0.89-1.22); 0.69
	Weighted median estimate	0.94 (0.81-1.10); 0.46	0.94 (0.78-1.13); 0.49	0.90 (0.69-1.18); 0.46

ER+ = Oestrogen-receptor positive, ER- = Oestrogen-receptor negative, 95% CI = 95% Confidence Interval, PAI-1 = Plasminogen activator inhibitor-1.

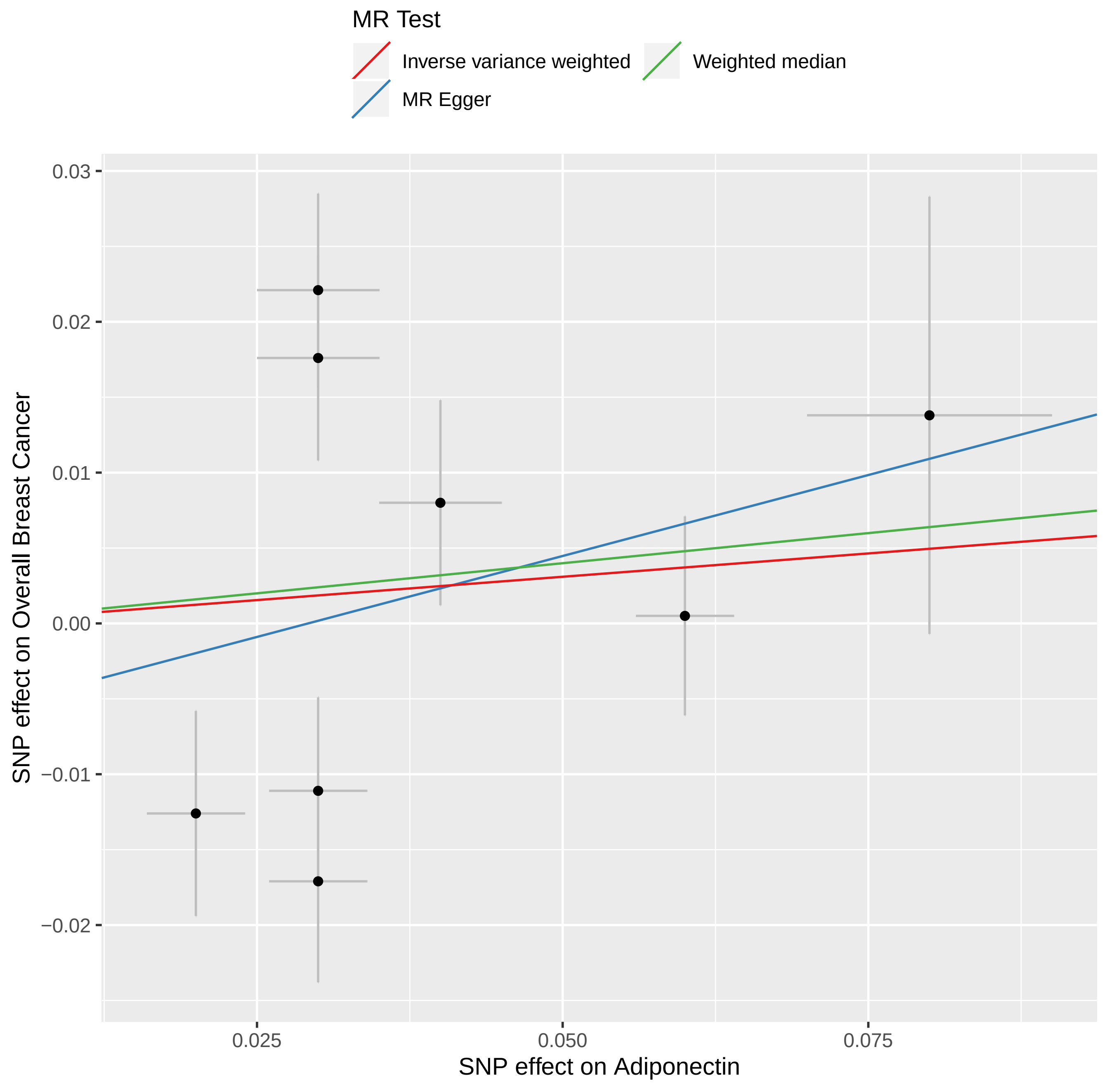
MR Test
Inverse variance weighted
Weighted median
MR Egger
0.03
0.02
0.01
0.00
−0.01
−0.02
SNP effect on Overall Breast Cancer
0.025
0.050
0.075
SNP effect on Adiponectin

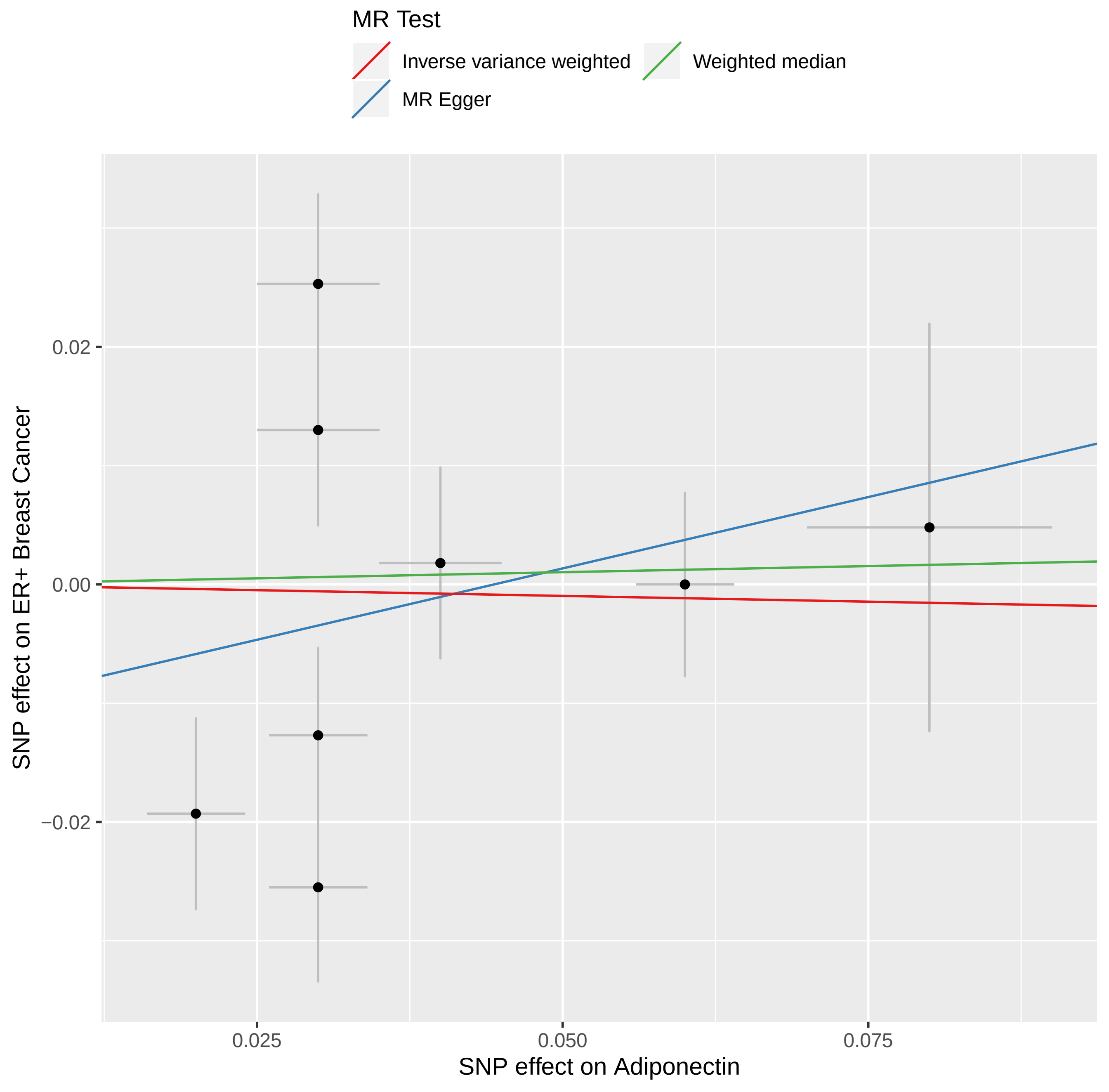
MR Test
Inverse variance weighted
Weighted median
MR Egger
0.02
0.00
−0.02
0.025
0.050
0.075
SNP effect on ER+ Breast Cancer
SNP effect on Adiponectin

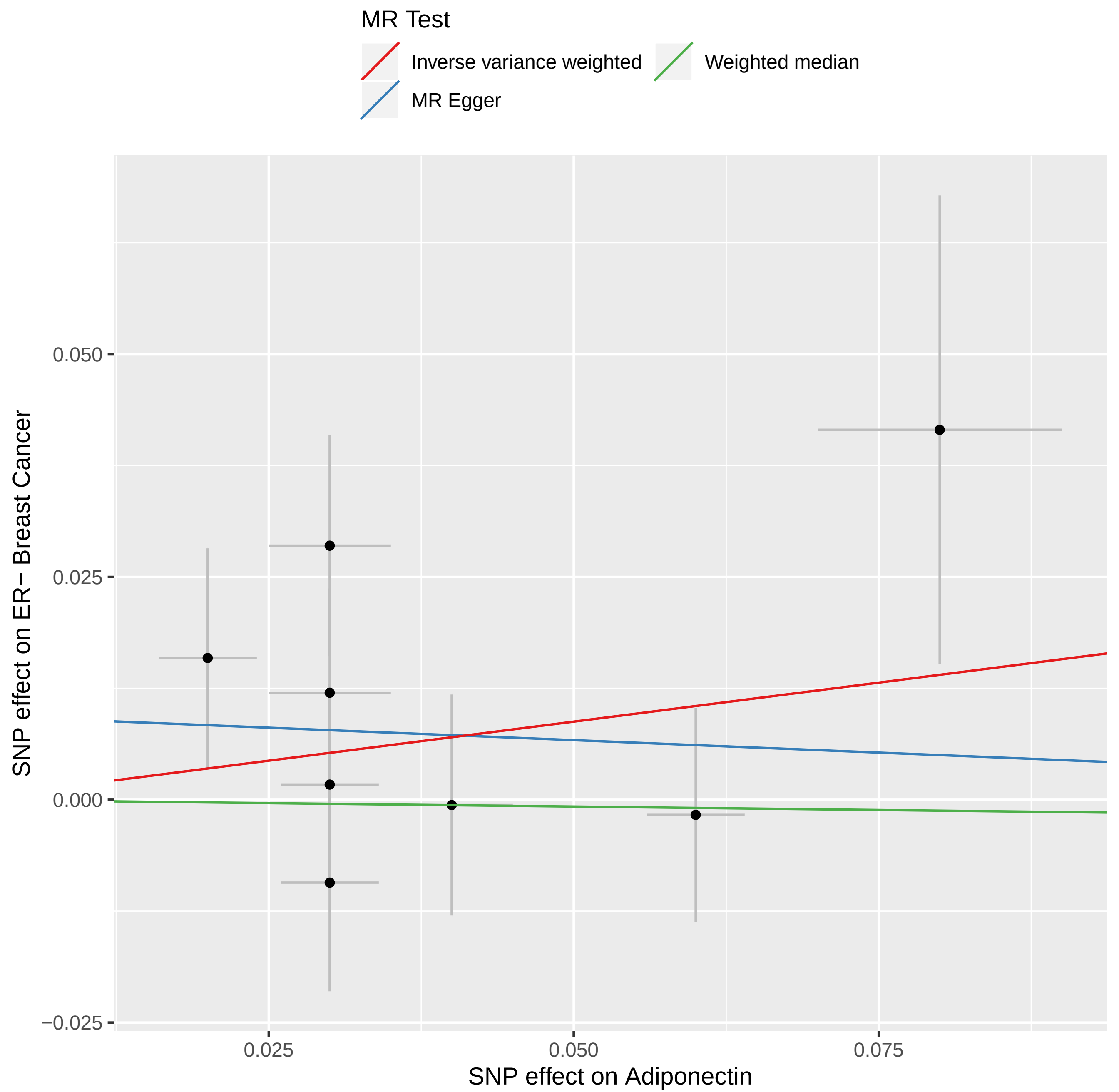
MR Test
Inverse variance weighted
Weighted median
MR Egger
0.050
0.025
0.000
−0.025
SNP effect on ER− Breast Cancer
0.025
0.050
0.075
SNP effect on Adiponectin

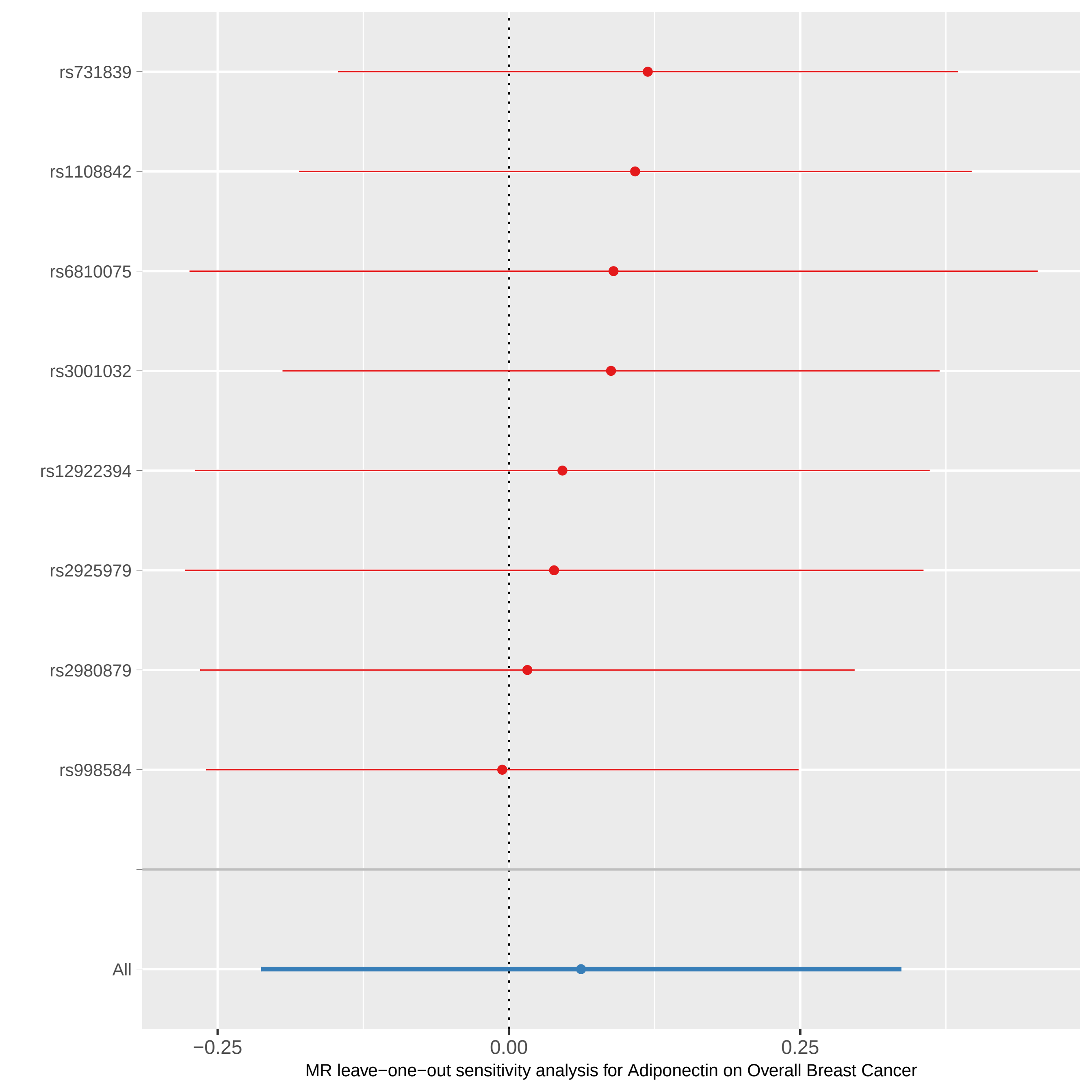
rs731839
rs1108842
rs6810075
rs3001032
rs12922394
rs2925979
rs2980879
rs998584
All
−0.25
0.00
0.25
MR leave−one−out sensitivity analysis for Adiponectin on Overall Breast Cancer

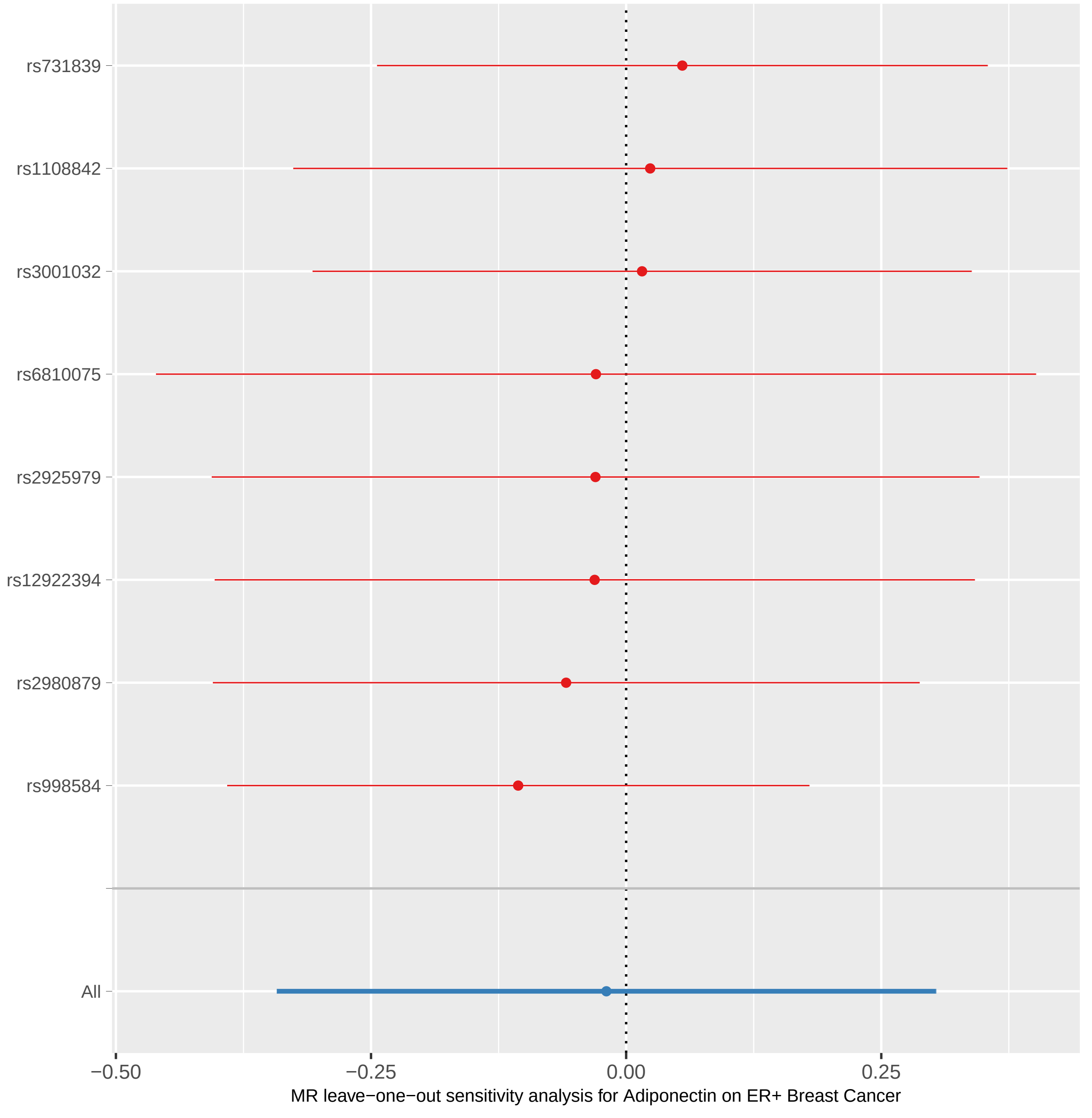
rs731839
rs1108842
rs3001032
rs6810075
rs2925979
rs12922394
rs2980879
rs998584
All
−0.50
−0.25
0.00
0.25
MR leave−one−out sensitivity analysis for Adiponectin on ER+ Breast Cancer

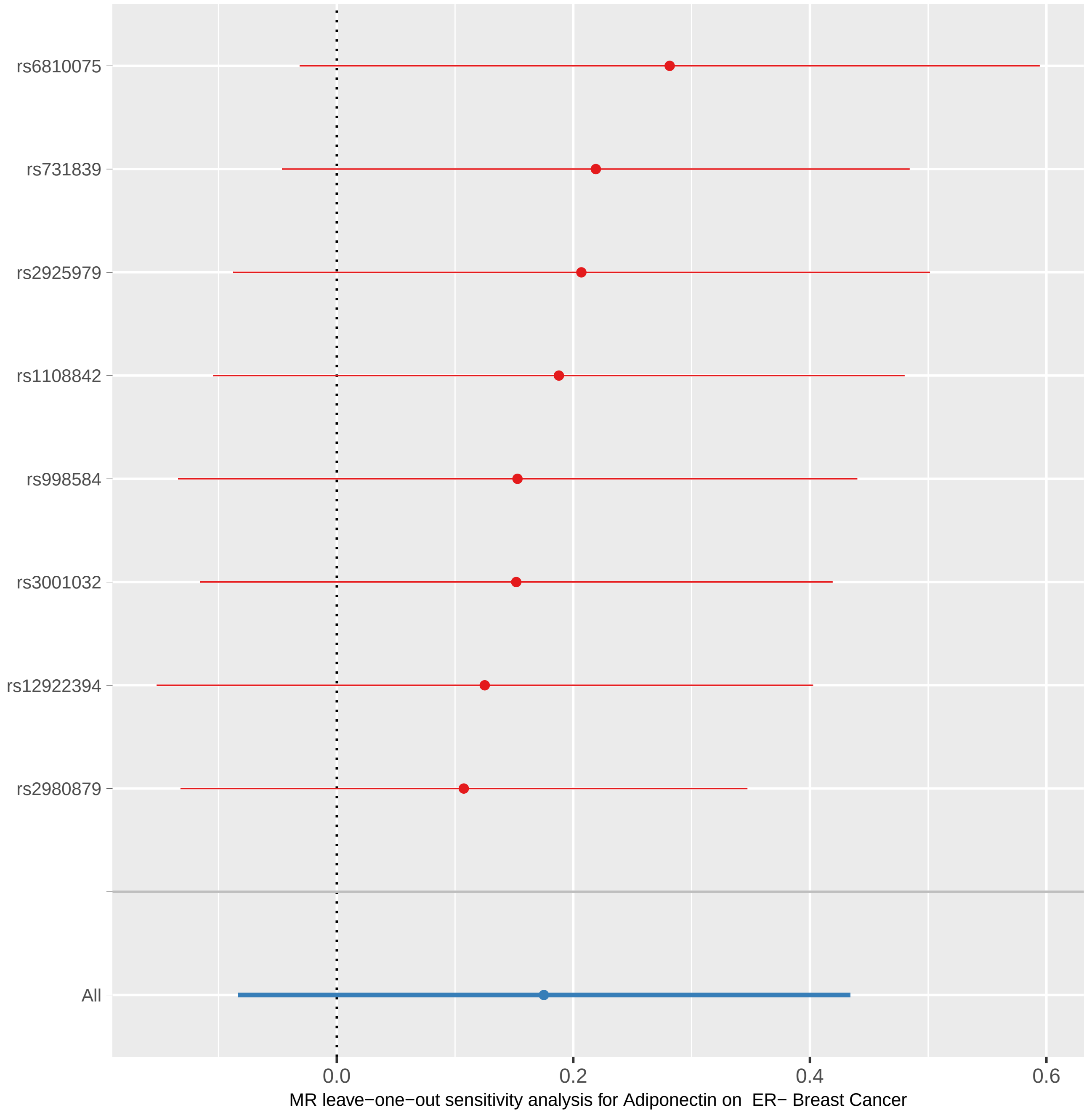
rs6810075
rs731839
rs2925979
rs1108842
rs998584
rs3001032
rs12922394
rs2980879
All
0.0
0.2
0.4
0.6
MR leave−one−out sensitivity analysis for Adiponectin on ER− Breast Cancer

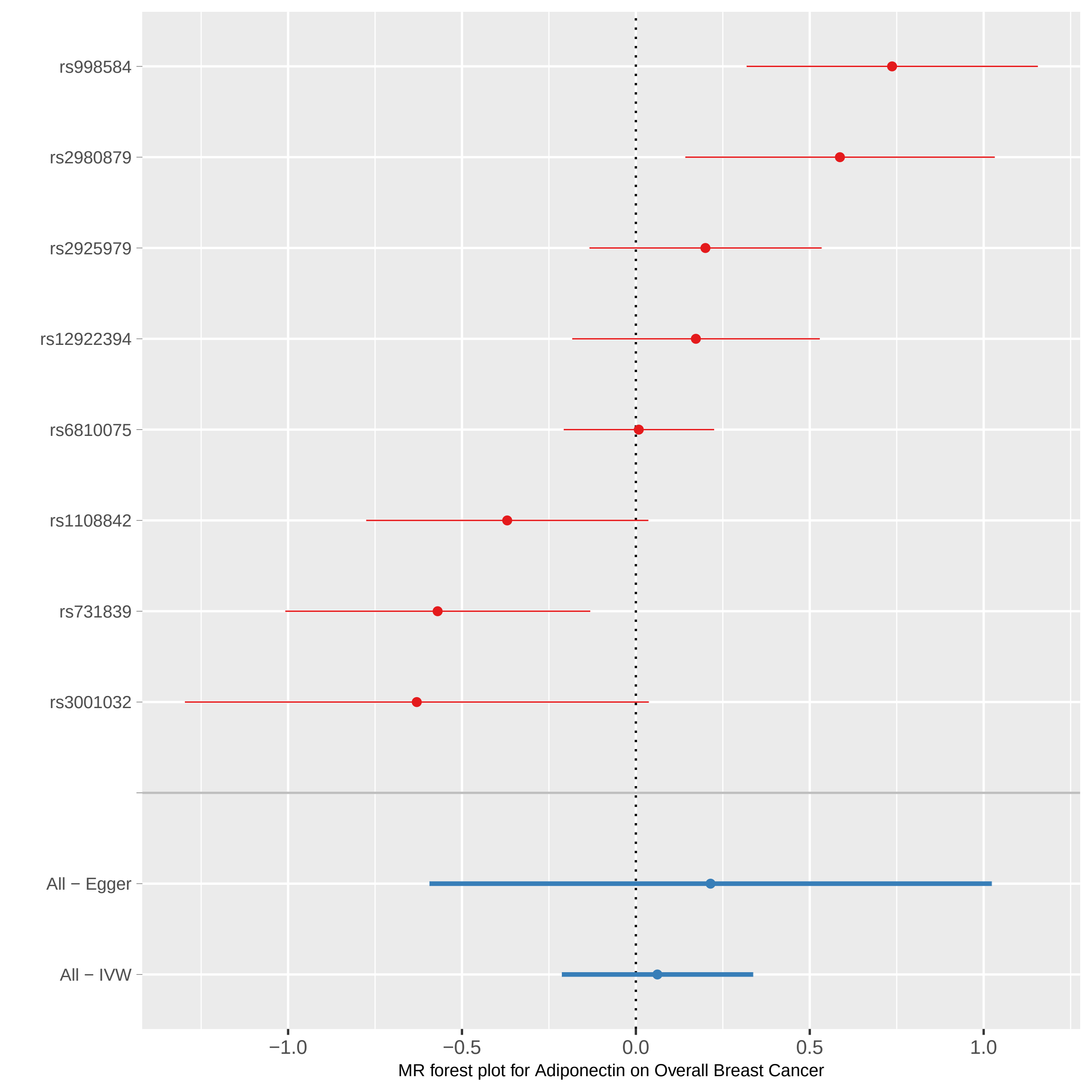

rs998584
rs2980879
rs2925979
rs12922394
rs6810075
rs1108842
rs731839
rs3001032
All − Egger
All − IVW
−1.0
−0.5
0.0
0.5
1.0
MR forest plot for Adiponectin on Overall Breast Cancer

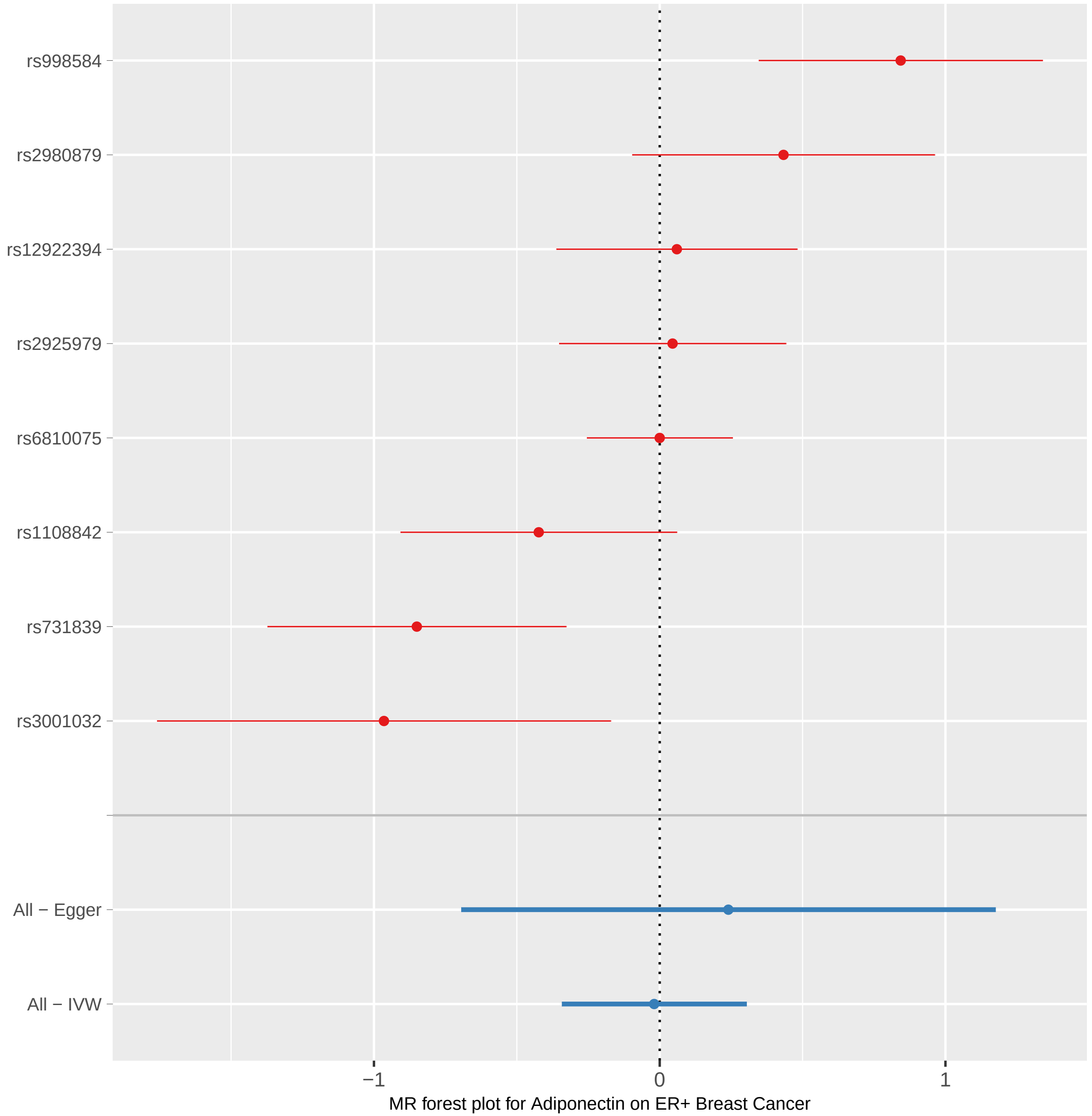

rs998584
rs2980879
rs12922394
rs2925979
rs6810075
rs1108842
rs731839
rs3001032
All − Egger
All − IVW
−1
0
1
MR forest plot for Adiponectin on ER+ Breast Cancer

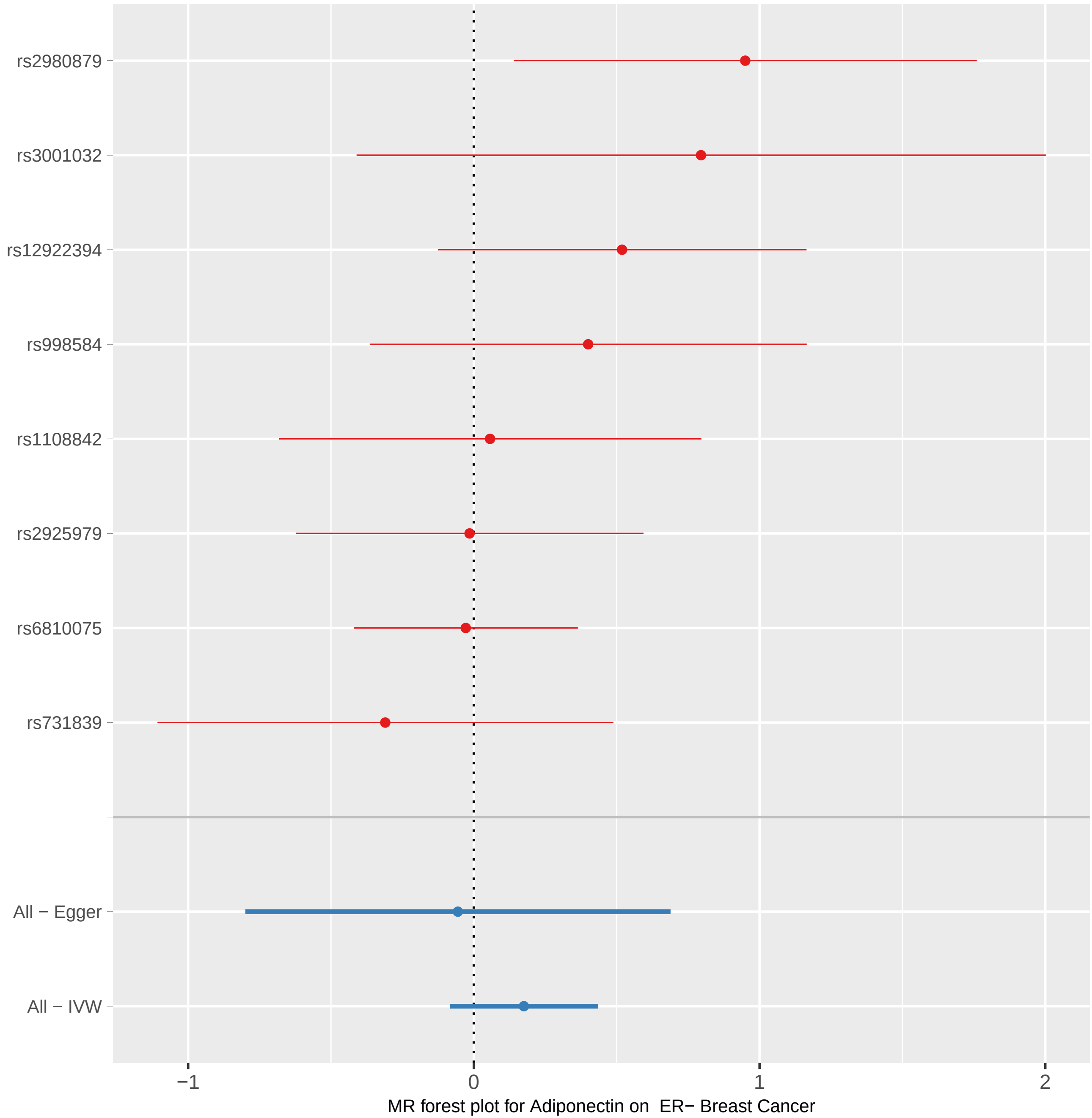
rs2980879
rs3001032
rs12922394
rs998584
rs1108842
rs2925979
rs6810075
rs731839
All − Egger
All − IVW
−1
0
1
2
MR forest plot for Adiponectin on ER− Breast Cancer

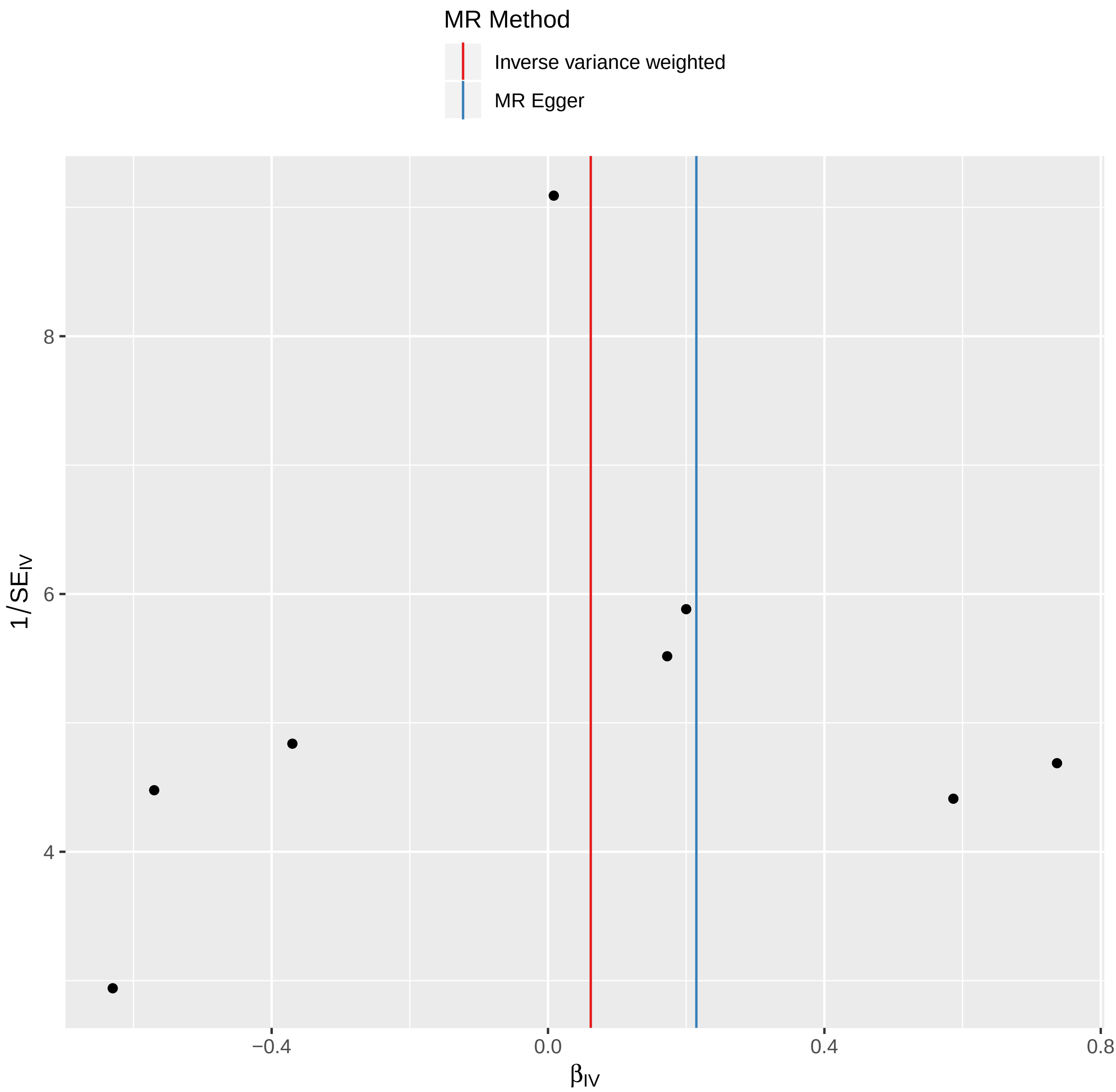

MR Method
Inverse variance weighted
MR Egger
$1/SE_{IV}$
8
6
4
−0.4
0.0
0.4
0.8
β_{IV}

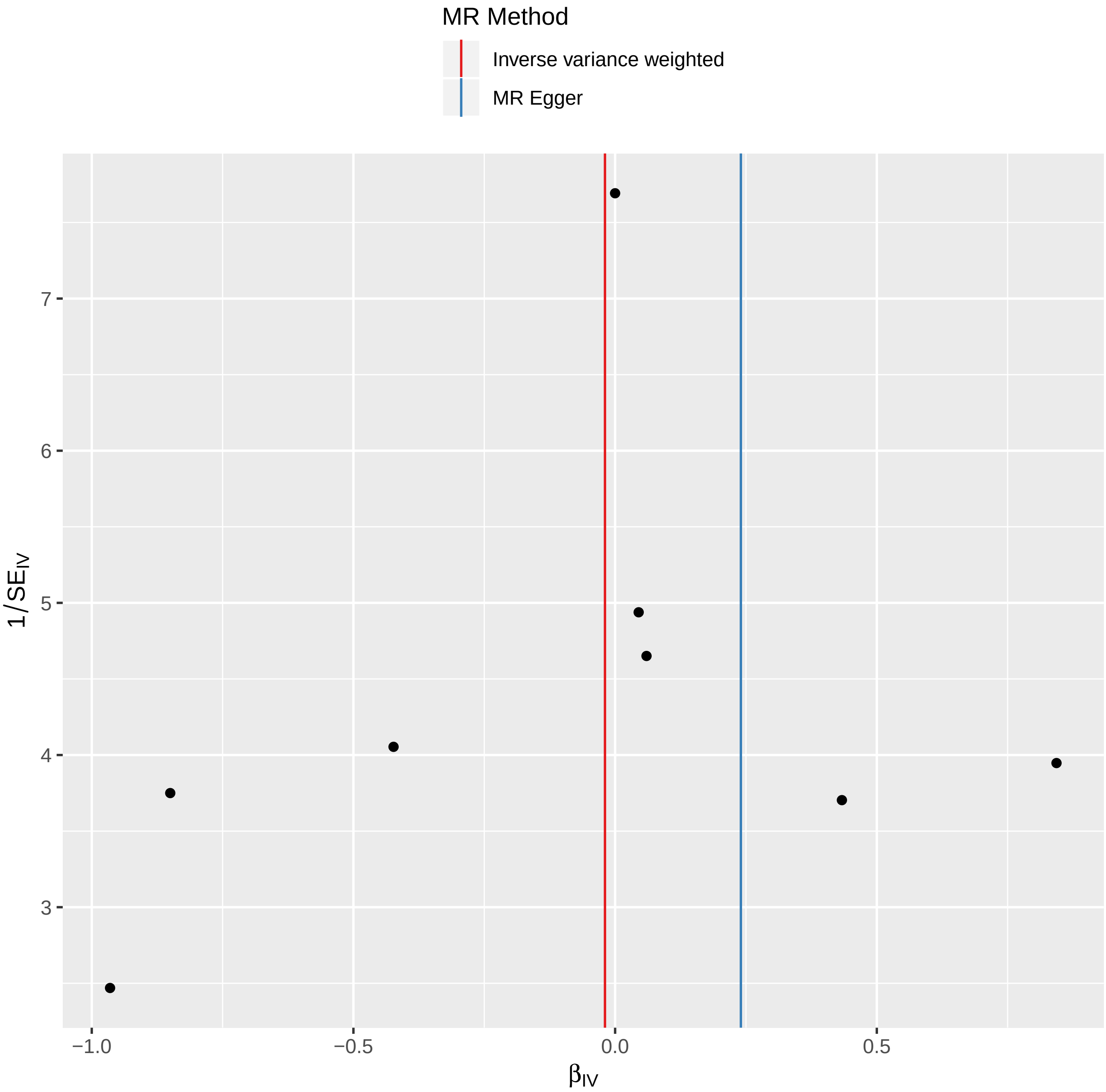
MR Method
Inverse variance weighted
MR Egger
7
6
5
4
3
1/SE_IV
−1.0
−0.5
0.0
0.5
β_IV

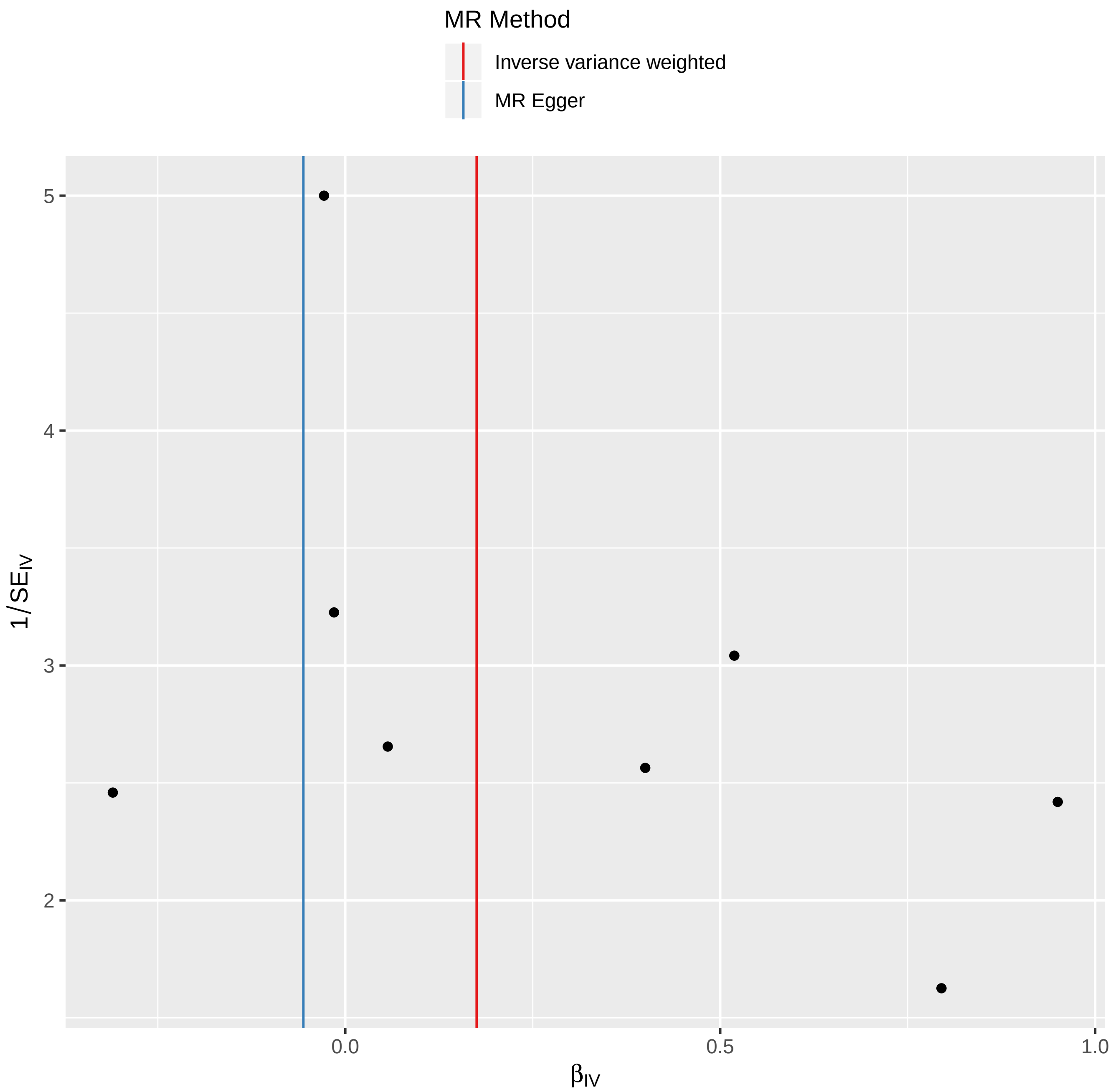
MR Method
Inverse variance weighted
MR Egger
1/SE_IV
5
4
3
2
0.0
0.5
1.0
β_IV

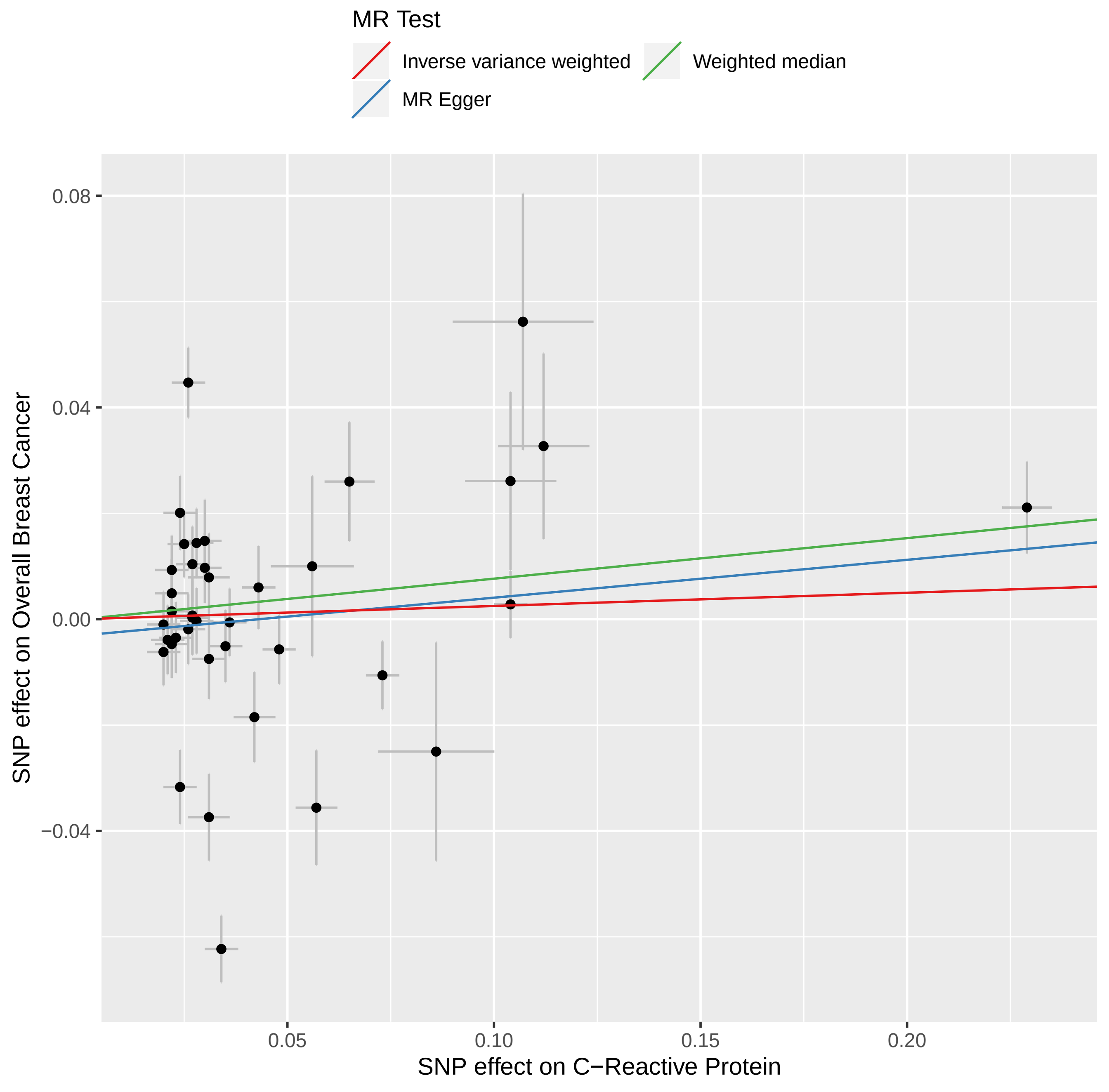
MR Test
Inverse variance weighted
Weighted median
MR Egger
SNP effect on Overall Breast Cancer
0.08
0.04
0.00
−0.04
0.05
0.10
0.15
0.20
SNP effect on C−Reactive Protein

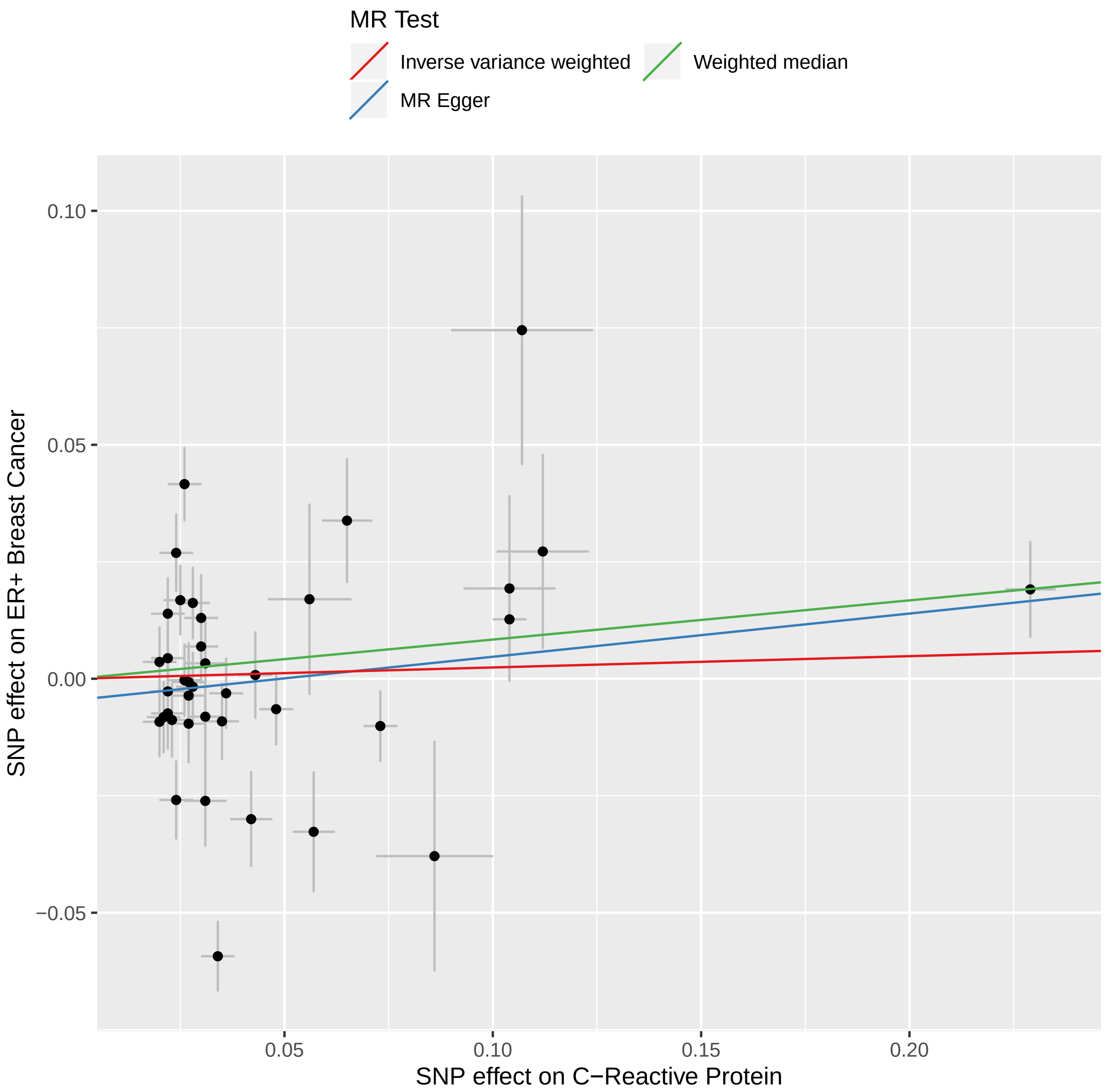
MR Test
Inverse variance weighted
Weighted median
MR Egger
0.10
0.05
0.00
−0.05
SNP effect on ER+ Breast Cancer
0.05
0.10
0.15
0.20
SNP effect on C−Reactive Protein

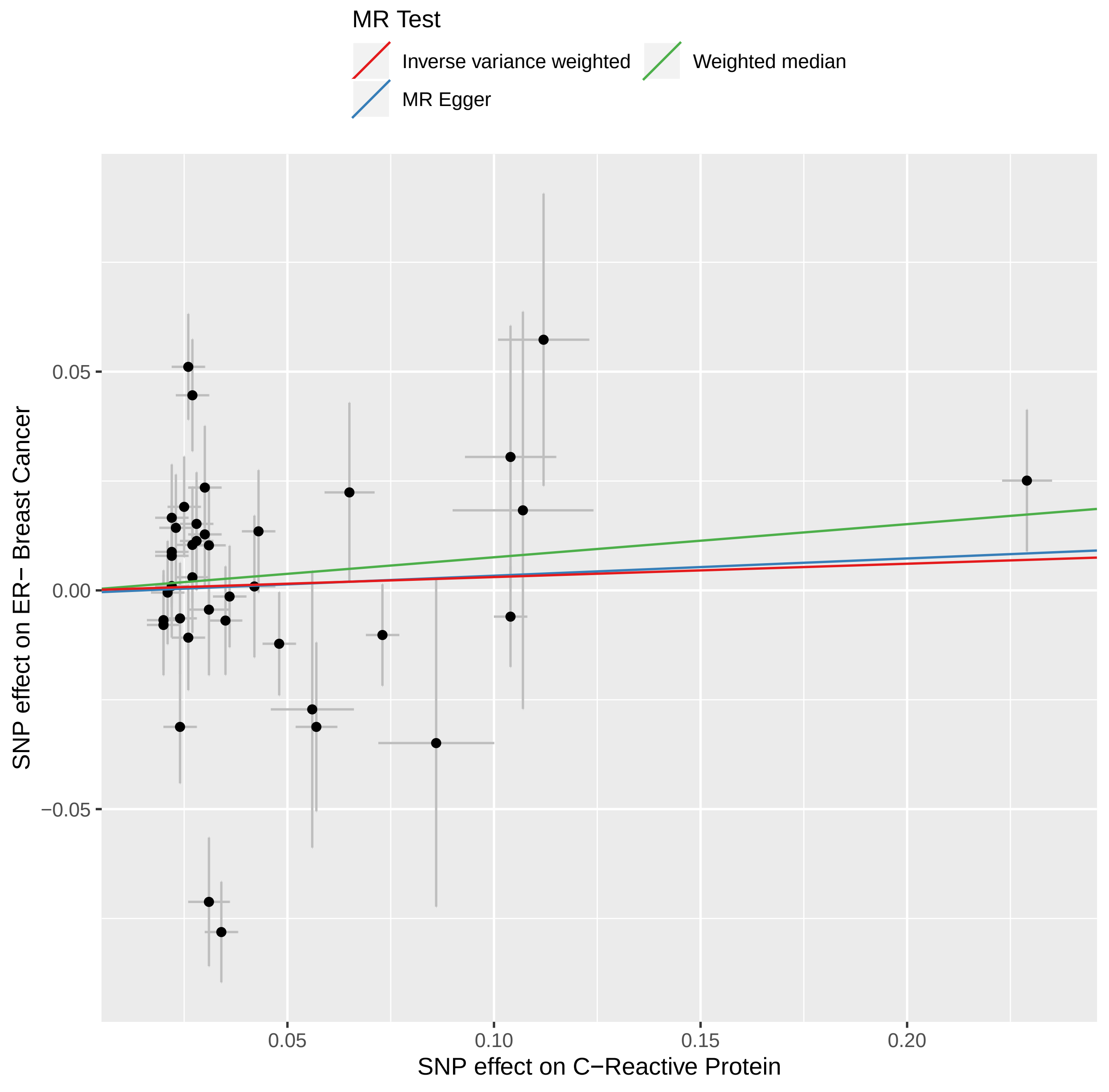
MR Test
Inverse variance weighted
MR Egger
Weighted median
0.05
0.00
−0.05
SNP effect on ER− Breast Cancer
0.05
0.10
0.15
0.20
SNP effect on C−Reactive Protein

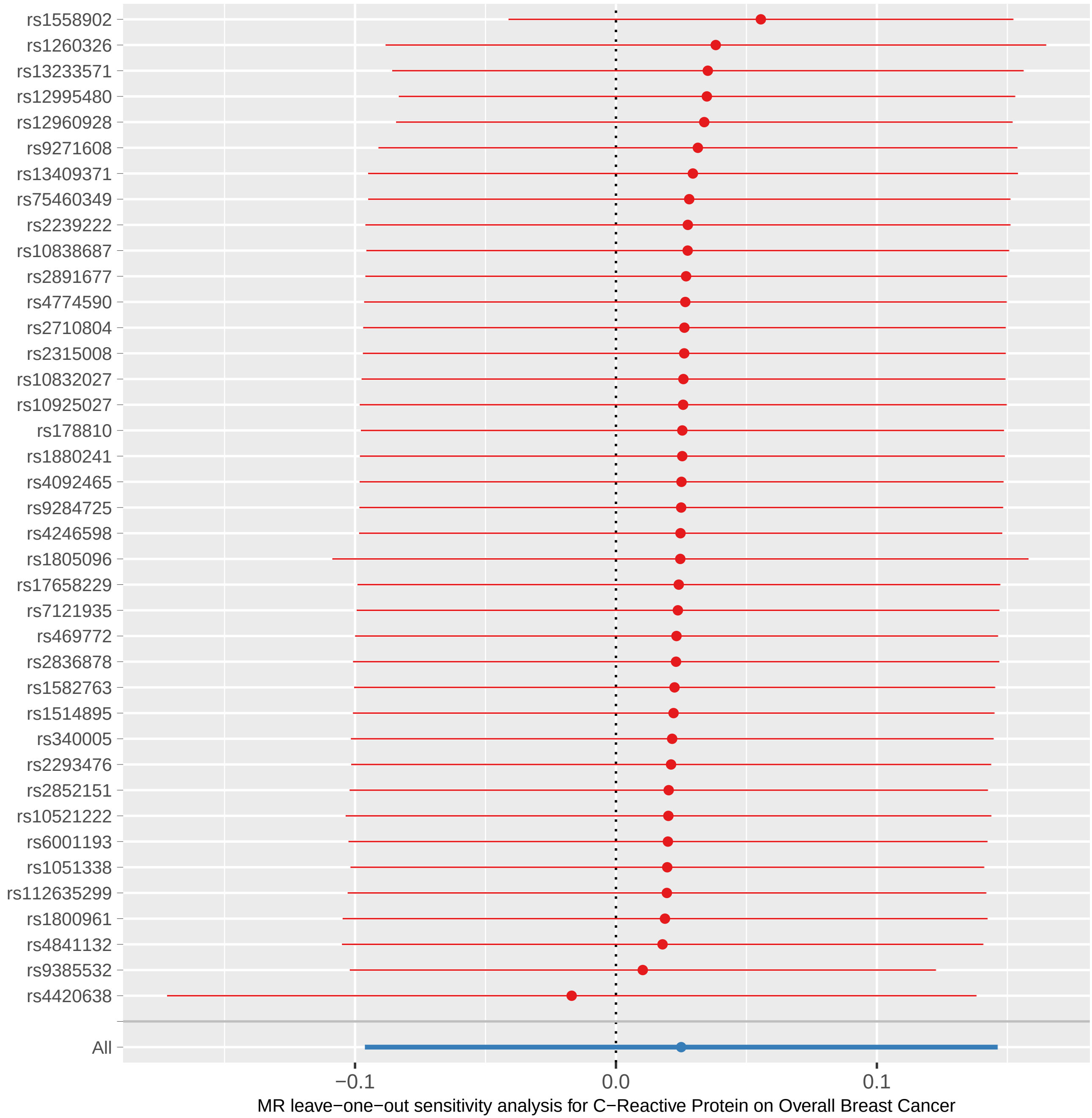
rs1558902
rs1260326
rs13233571
rs12995480
rs12960928
rs9271608
rs13409371
rs75460349
rs2239222
rs10838687
rs2891677
rs4774590
rs2710804
rs2315008
rs10832027
rs10925027
rs178810
rs1880241
rs4092465
rs9284725
rs4246598
rs1805096
rs17658229
rs7121935
rs469772
rs2836878
rs1582763
rs1514895
rs340005
rs2293476
rs2852151
rs10521222
rs6001193
rs1051338
rs112635299
rs1800961
rs4841132
rs9385532
rs4420638
All
−0.1
0.0
0.1
MR leave−one−out sensitivity analysis for C−Reactive Protein on Overall Breast Cancer

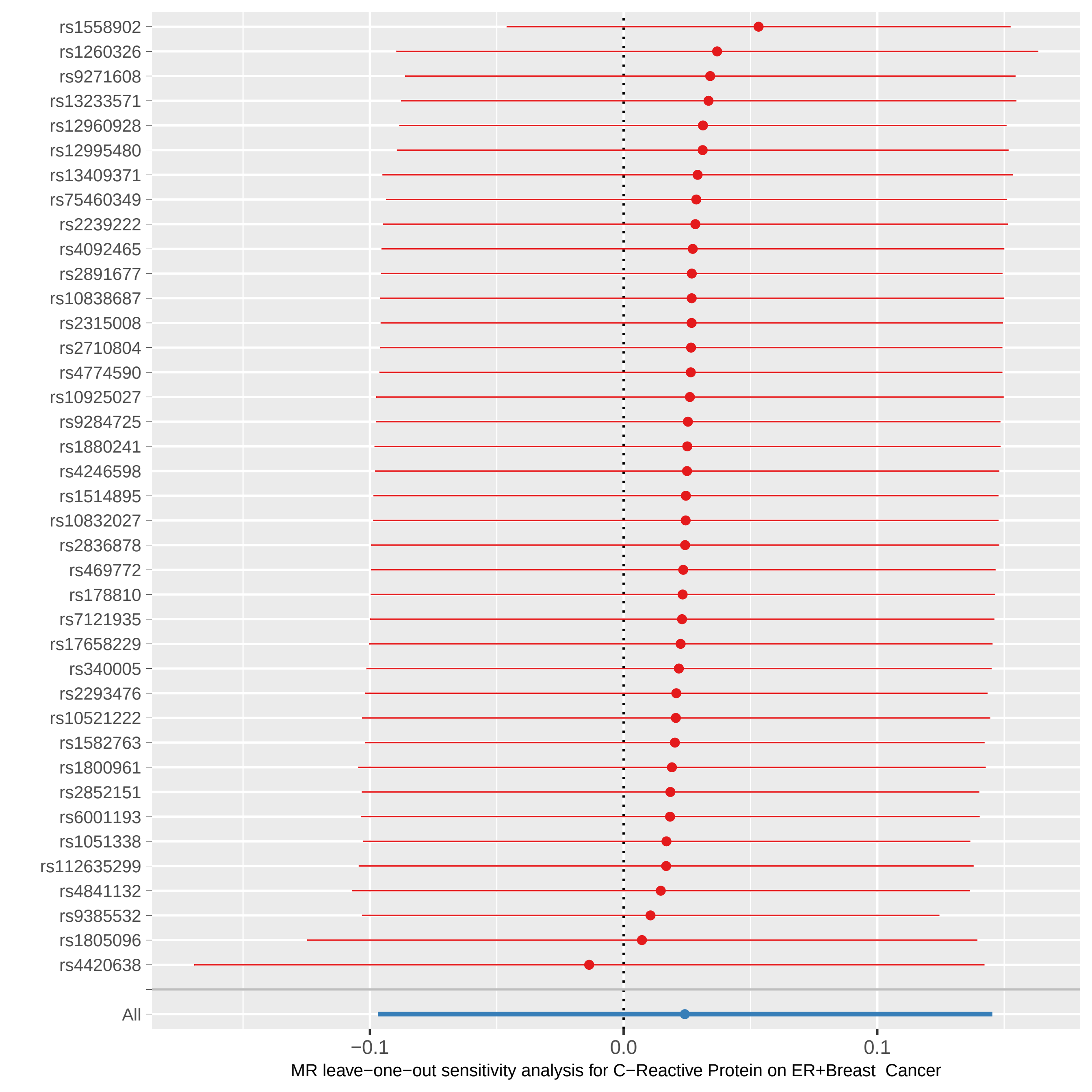
rs1558902
rs1260326
rs9271608
rs13233571
rs12960928
rs12995480
rs13409371
rs75460349
rs2239222
rs4092465
rs2891677
rs10838687
rs2315008
rs2710804
rs4774590
rs10925027
rs9284725
rs1880241
rs4246598
rs1514895
rs10832027
rs2836878
rs469772
rs178810
rs7121935
rs17658229
rs340005
rs2293476
rs10521222
rs1582763
rs1800961
rs2852151
rs6001193
rs1051338
rs112635299
rs4841132
rs9385532
rs1805096
rs4420638
All
−0.1
0.0
0.1
MR leave−one−out sensitivity analysis for C−Reactive Protein on ER+Breast Cancer

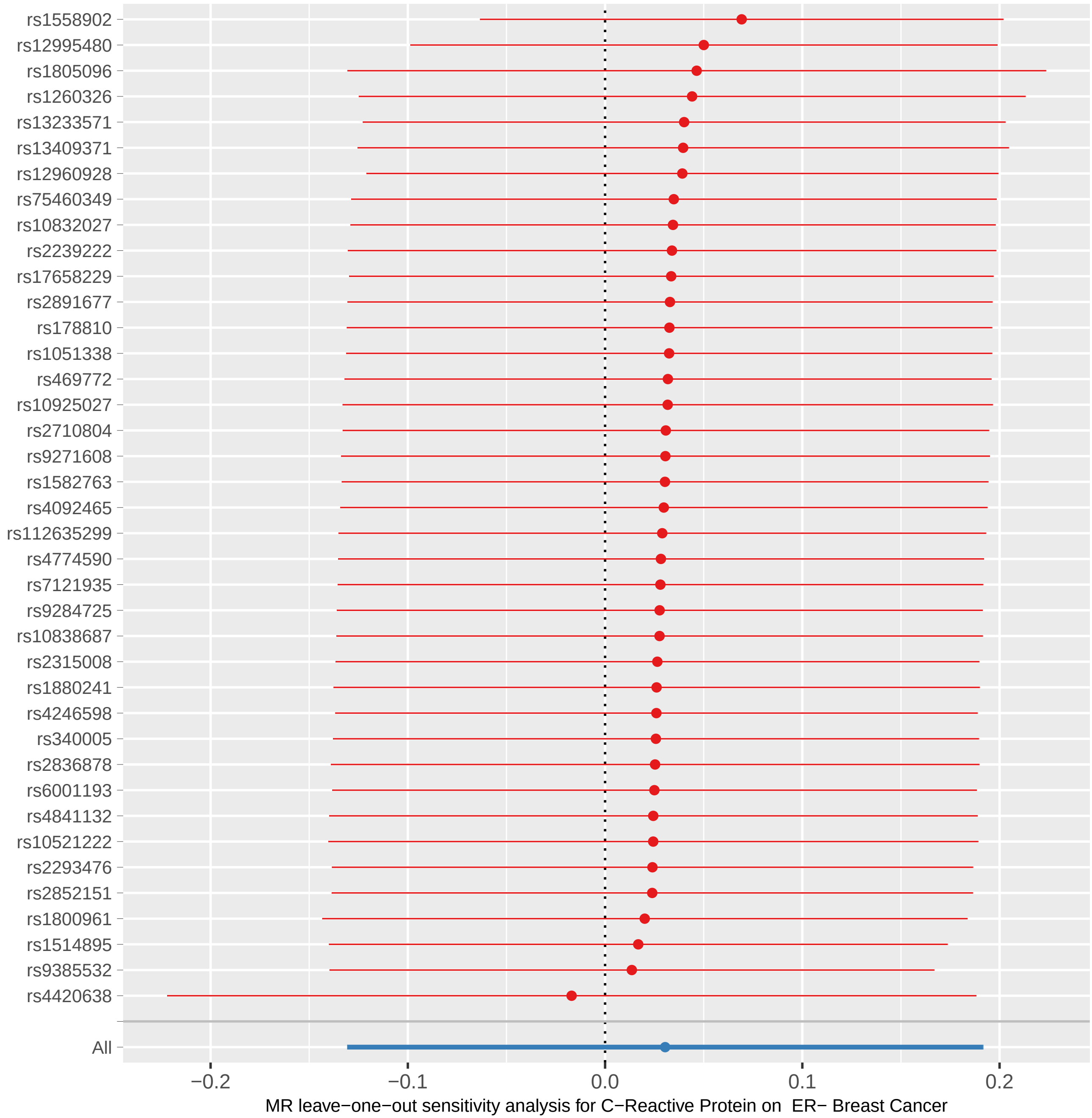

rs1558902
rs12995480
rs1805096
rs1260326
rs13233571
rs13409371
rs12960928
rs75460349
rs10832027
rs2239222
rs17658229
rs2891677
rs178810
rs1051338
rs469772
rs10925027
rs2710804
rs9271608
rs1582763
rs4092465
rs112635299
rs4774590
rs7121935
rs9284725
rs10838687
rs2315008
rs1880241
rs4246598
rs340005
rs2836878
rs6001193
rs4841132
rs10521222
rs2293476
rs2852151
rs1800961
rs1514895
rs9385532
rs4420638
All
−0.2
−0.1
0.0
0.1
0.2
MR leave−one−out sensitivity analysis for C−Reactive Protein on ER− Breast Cancer

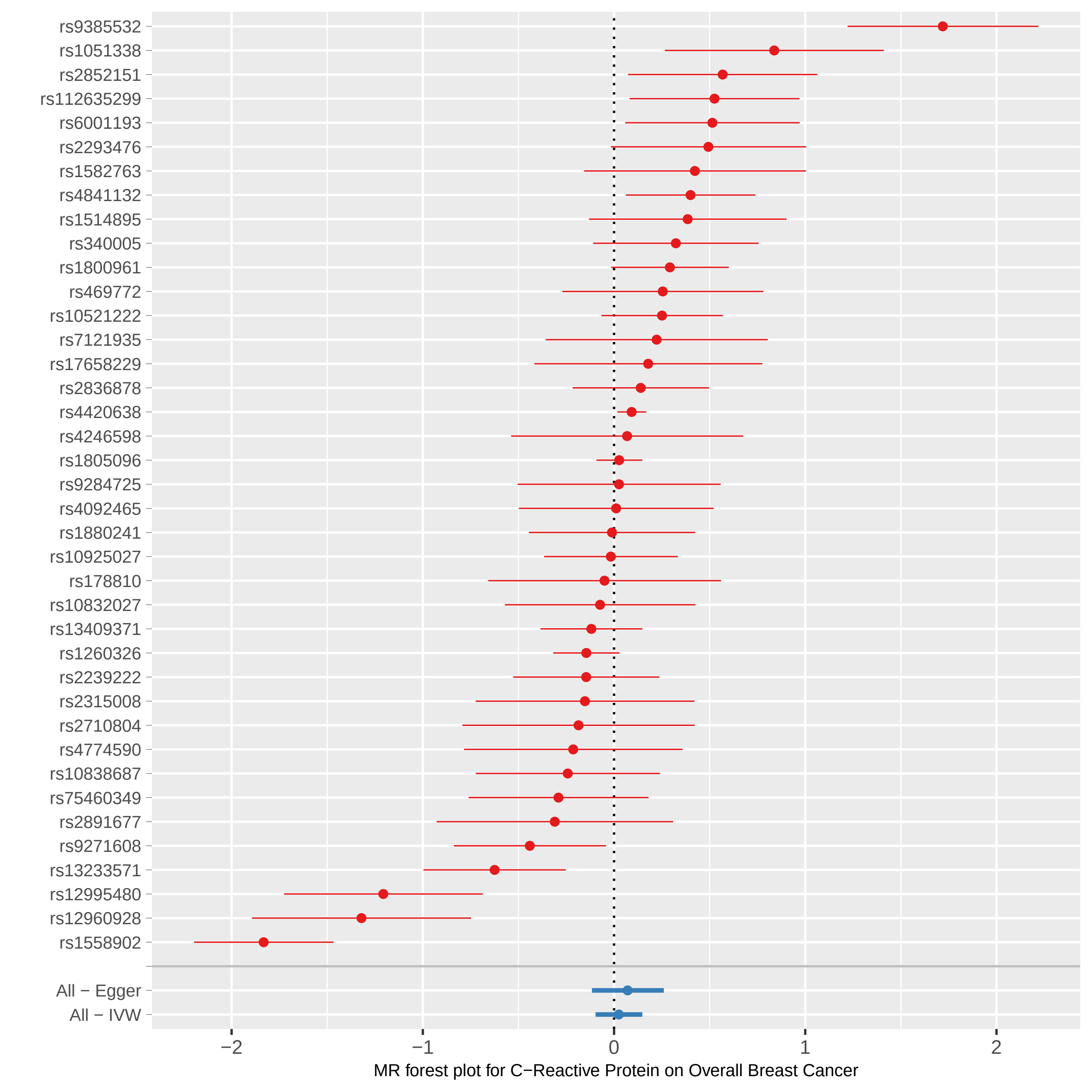
rs9385532
rs1051338
rs2852151
rs112635299
rs6001193
rs2293476
rs1582763
rs4841132
rs1514895
rs340005
rs1800961
rs469772
rs10521222
rs7121935
rs17658229
rs2836878
rs4420638
rs4246598
rs1805096
rs9284725
rs4092465
rs1880241
rs10925027
rs178810
rs10832027
rs13409371
rs1260326
rs2239222
rs2315008
rs2710804
rs4774590
rs10838687
rs75460349
rs2891677
rs9271608
rs13233571
rs12995480
rs12960928
rs1558902
All − Egger
All − IVW
−2
−1
0
1
2
MR forest plot for C−Reactive Protein on Overall Breast Cancer

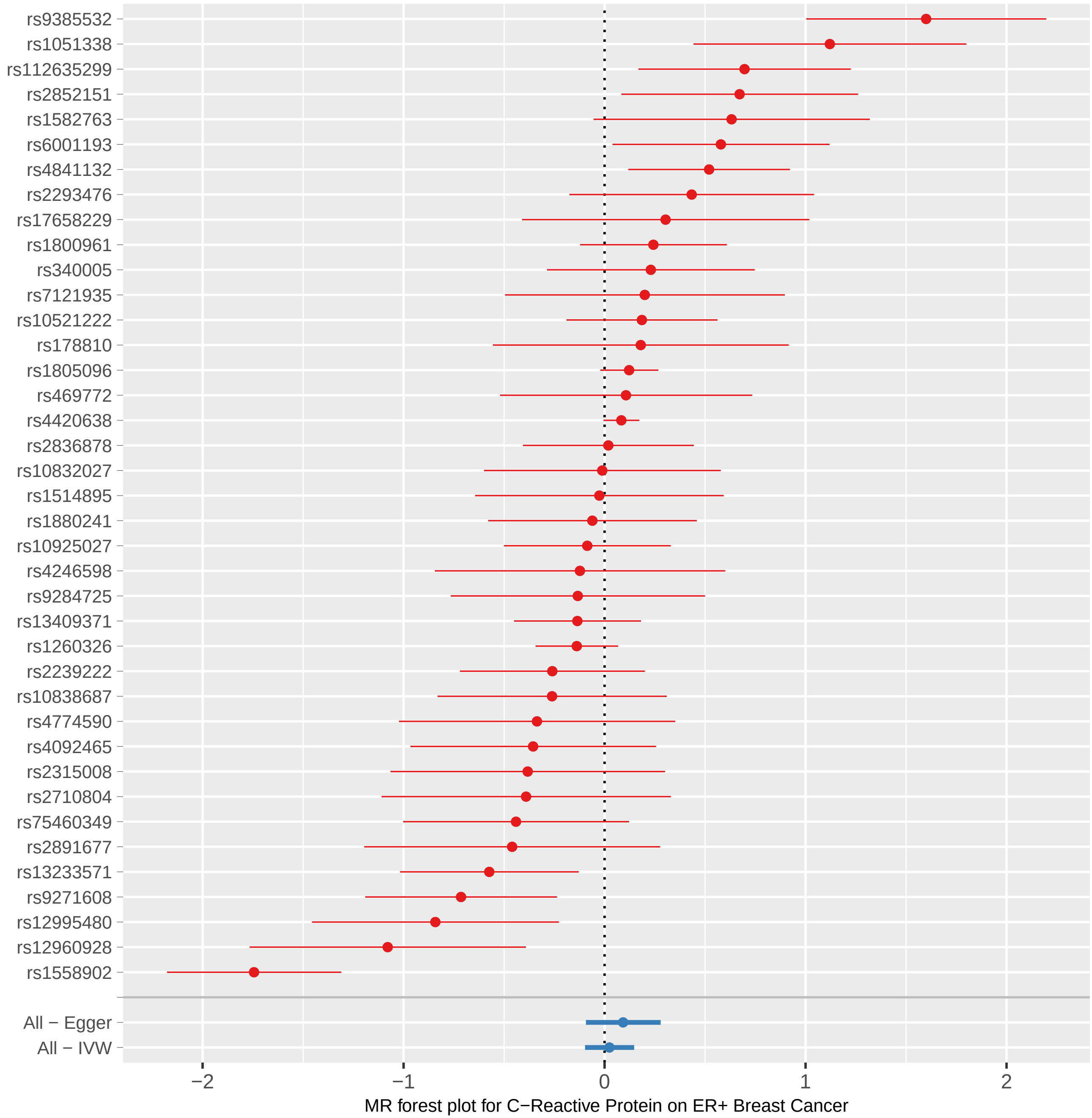

rs9385532
rs1051338
rs112635299
rs2852151
rs1582763
rs6001193
rs4841132
rs2293476
rs17658229
rs1800961
rs340005
rs7121935
rs10521222
rs178810
rs1805096
rs469772
rs4420638
rs2836878
rs10832027
rs1514895
rs1880241
rs10925027
rs4246598
rs9284725
rs13409371
rs1260326
rs2239222
rs10838687
rs4774590
rs4092465
rs2315008
rs2710804
rs75460349
rs2891677
rs13233571
rs9271608
rs12995480
rs12960928
rs1558902
All − Egger
All − IVW
−2
−1
0
1
2
MR forest plot for C−Reactive Protein on ER+ Breast Cancer

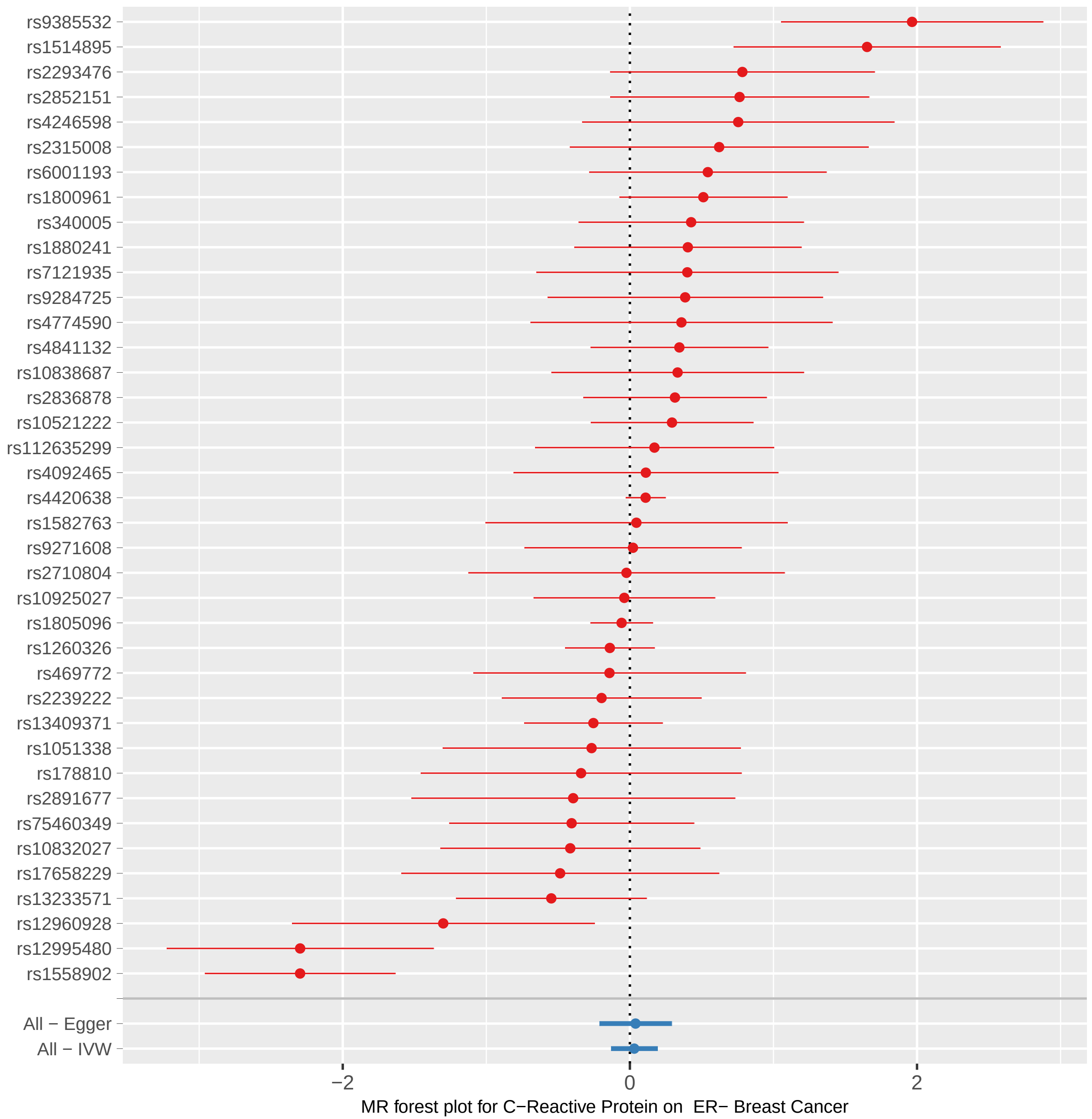
rs9385532
rs1514895
rs2293476
rs2852151
rs4246598
rs2315008
rs6001193
rs1800961
rs340005
rs1880241
rs7121935
rs9284725
rs4774590
rs4841132
rs10838687
rs2836878
rs10521222
rs112635299
rs4092465
rs4420638
rs1582763
rs9271608
rs2710804
rs10925027
rs1805096
rs1260326
rs469772
rs2239222
rs13409371
rs1051338
rs178810
rs2891677
rs75460349
rs10832027
rs17658229
rs13233571
rs12960928
rs12995480
rs1558902
All − Egger
All − IVW
−2
0
2
MR forest plot for C−Reactive Protein on ER− Breast Cancer

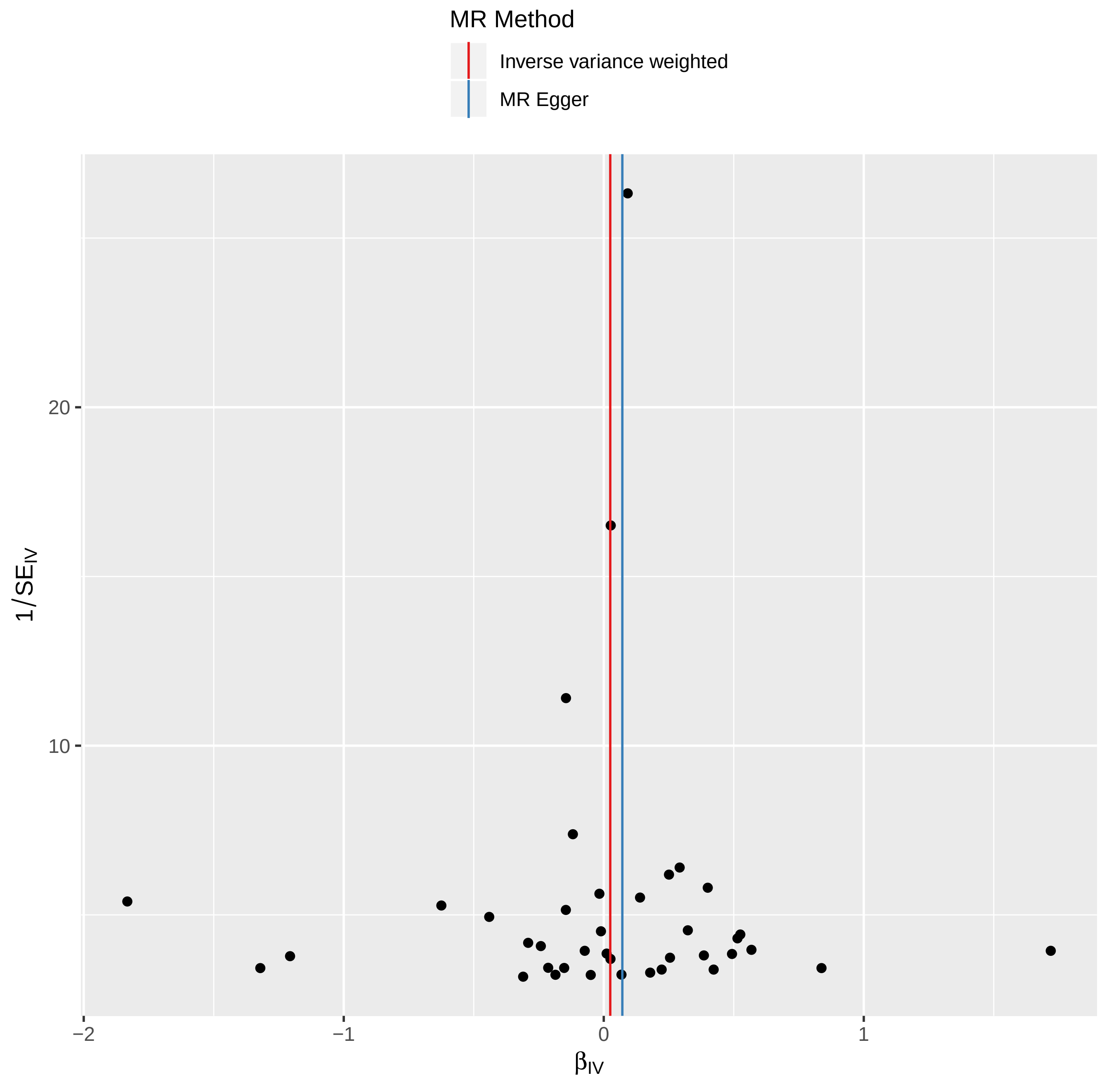

MR Method
Inverse variance weighted
MR Egger
20
10
1/SE$_{IV}$
−2
−1
0
1
β$_{IV}$

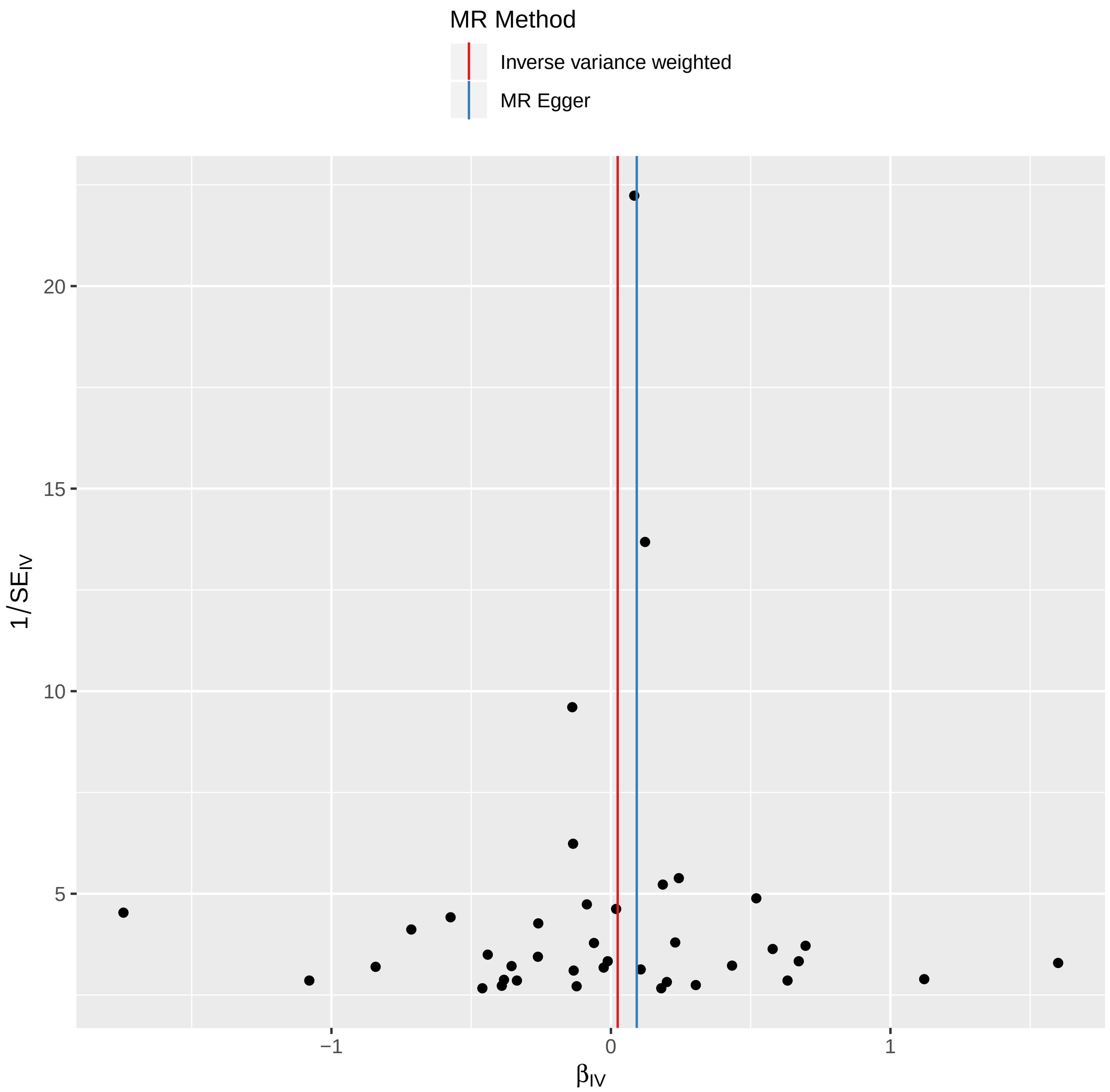
MR Method
Inverse variance weighted
MR Egger
20
15
10
5
1/SE_IV
−1
0
1
β_IV

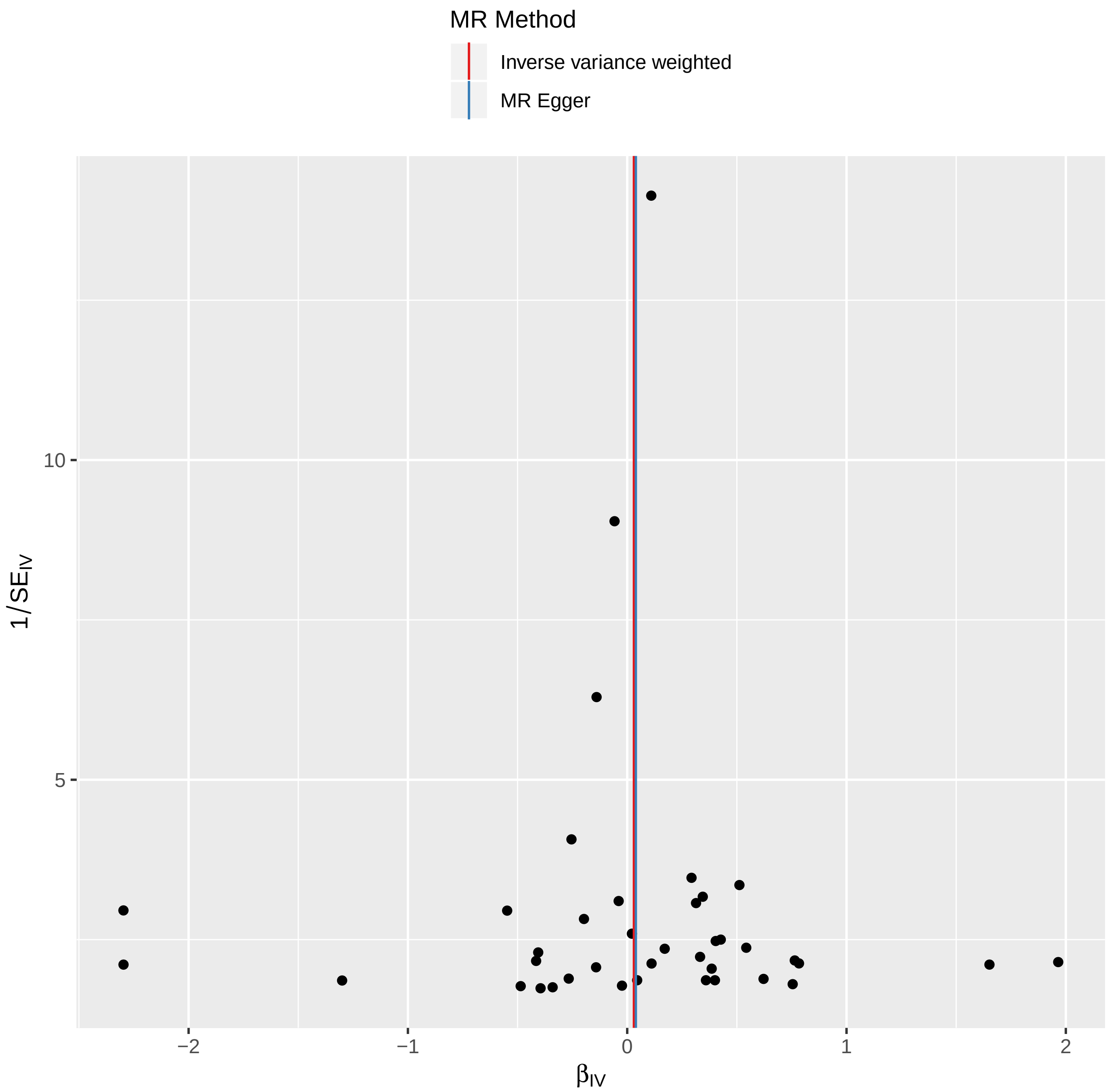
MR Method
Inverse variance weighted
MR Egger
10
5
1/SE_IV
−2
−1
0
1
2
β_IV

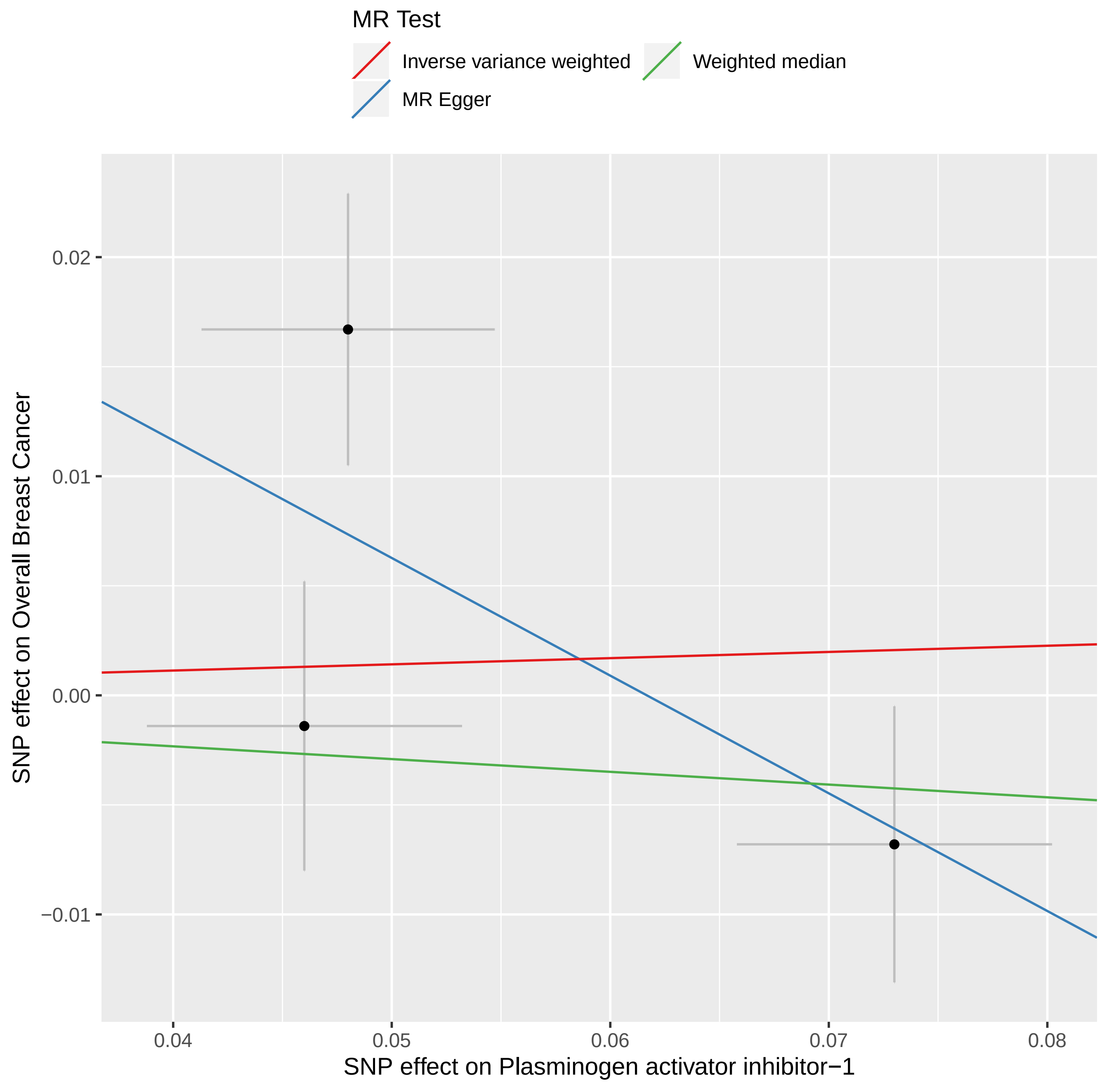

MR Test
Inverse variance weighted
Weighted median
MR Egger
0.02
0.01
0.00
−0.01
0.04
0.05
0.06
0.07
0.08
SNP effect on Overall Breast Cancer
SNP effect on Plasminogen activator inhibitor−1

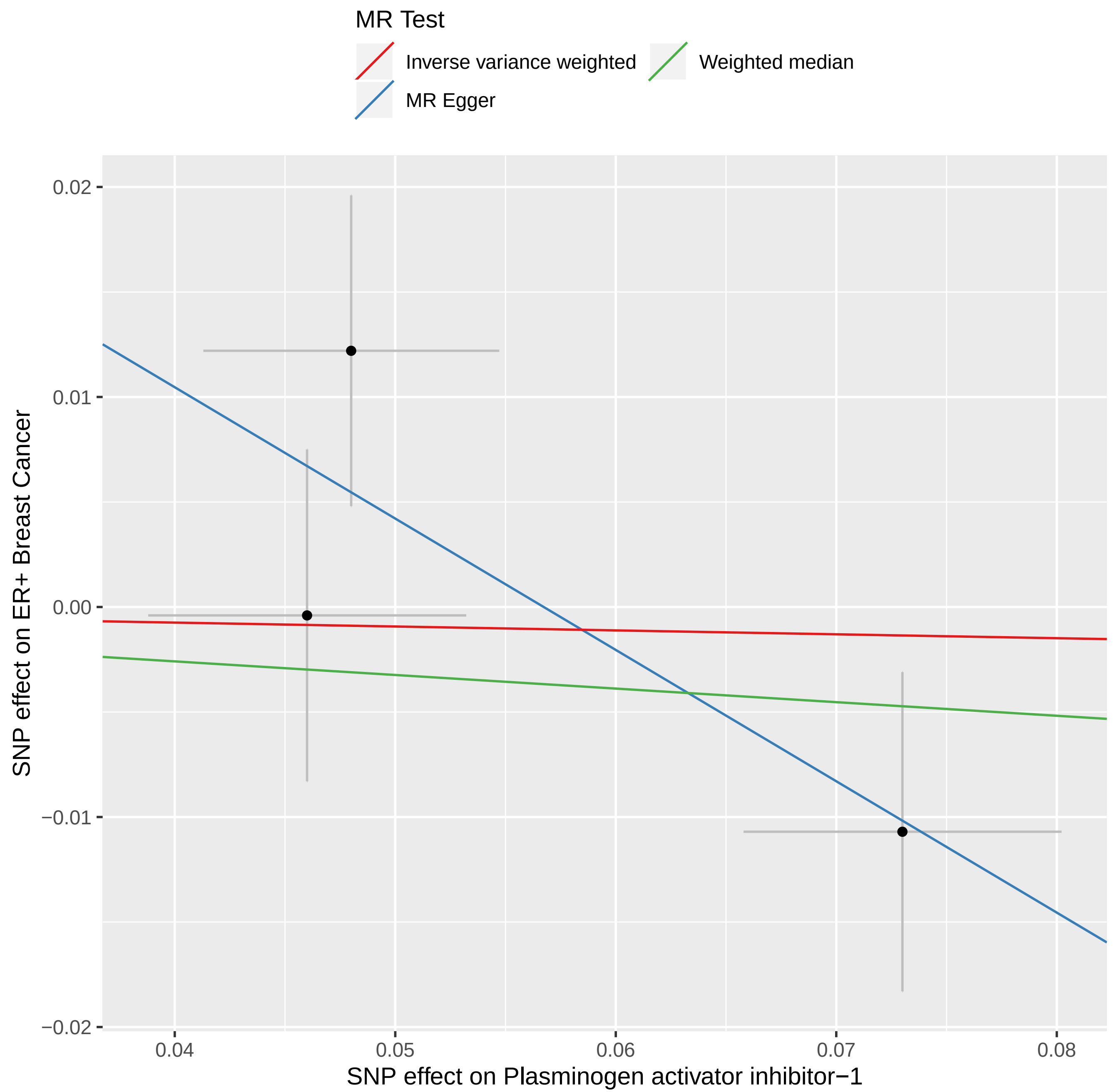

MR Test
Inverse variance weighted
Weighted median
MR Egger
0.02
0.01
0.00
−0.01
−0.02
0.04
0.05
0.06
0.07
0.08
SNP effect on ER+ Breast Cancer
SNP effect on Plasminogen activator inhibitor−1

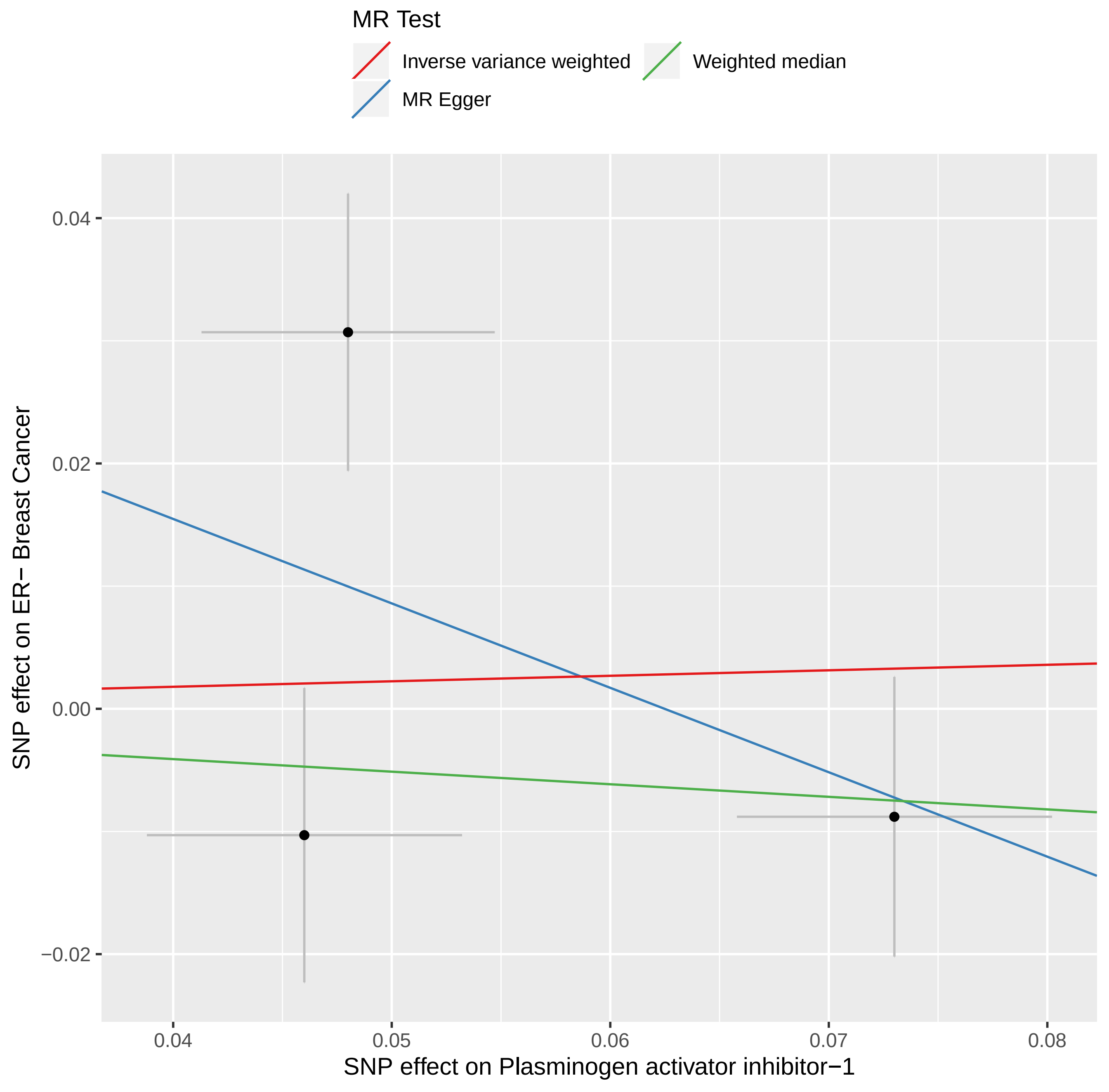
MR Test
Inverse variance weighted
Weighted median
MR Egger
0.04
0.02
0.00
−0.02
0.04
0.05
0.06
0.07
0.08
SNP effect on ER− Breast Cancer
SNP effect on Plasminogen activator inhibitor−1

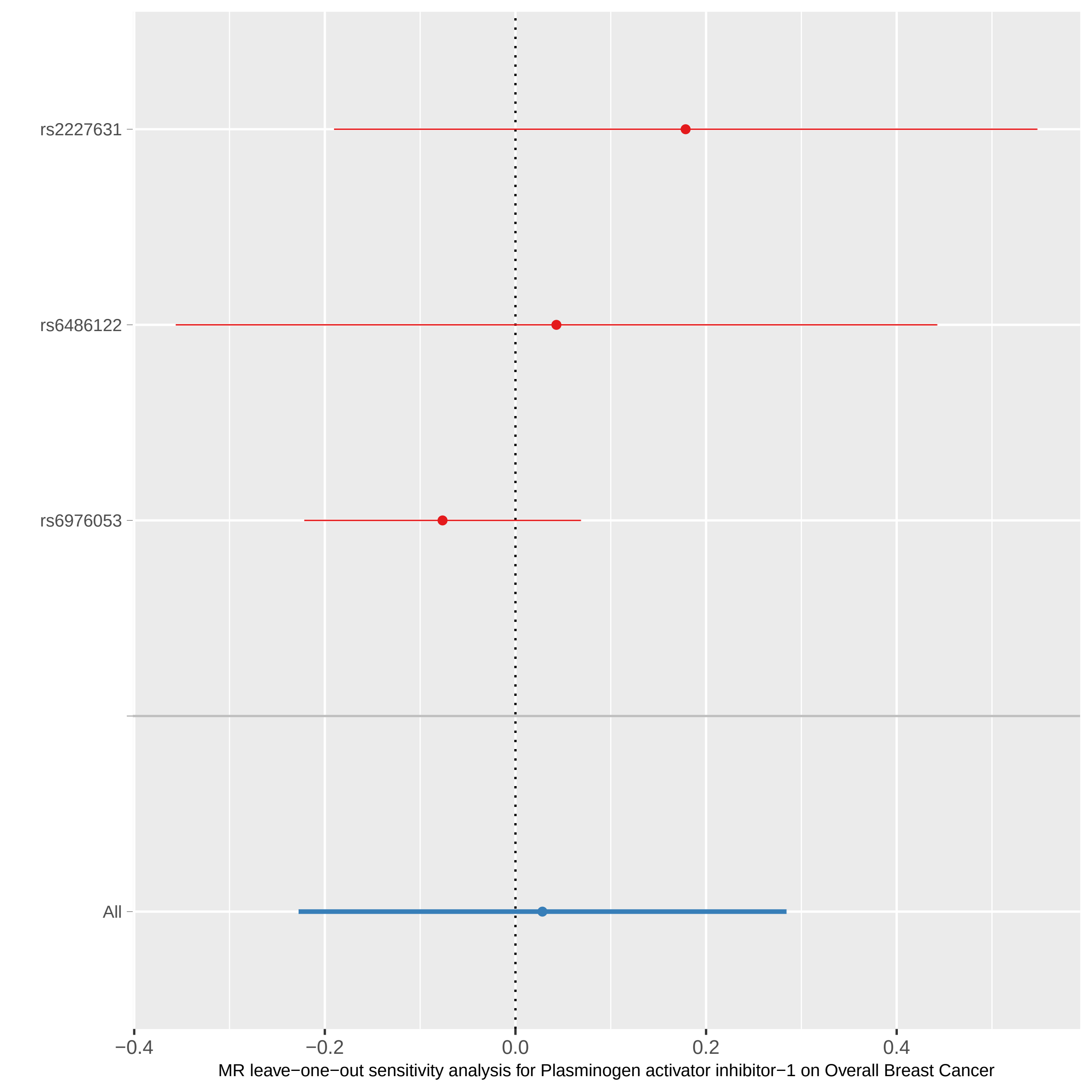
rs2227631
rs6486122
rs6976053
All
−0.4
−0.2
0.0
0.2
0.4
MR leave−one−out sensitivity analysis for Plasminogen activator inhibitor−1 on Overall Breast Cancer

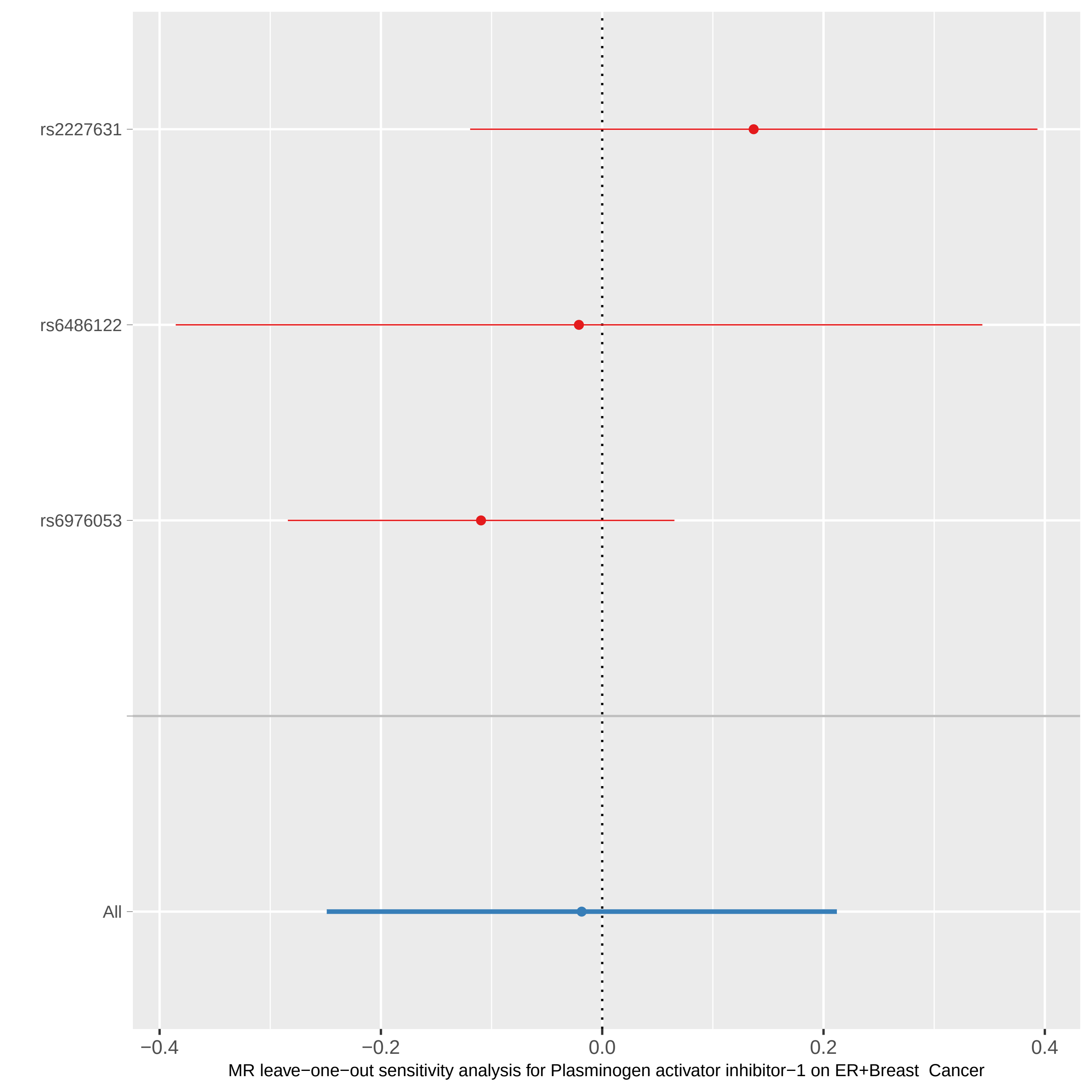
rs2227631
rs6486122
rs6976053
All
−0.4
−0.2
0.0
0.2
0.4
MR leave−one−out sensitivity analysis for Plasminogen activator inhibitor−1 on ER+Breast Cancer

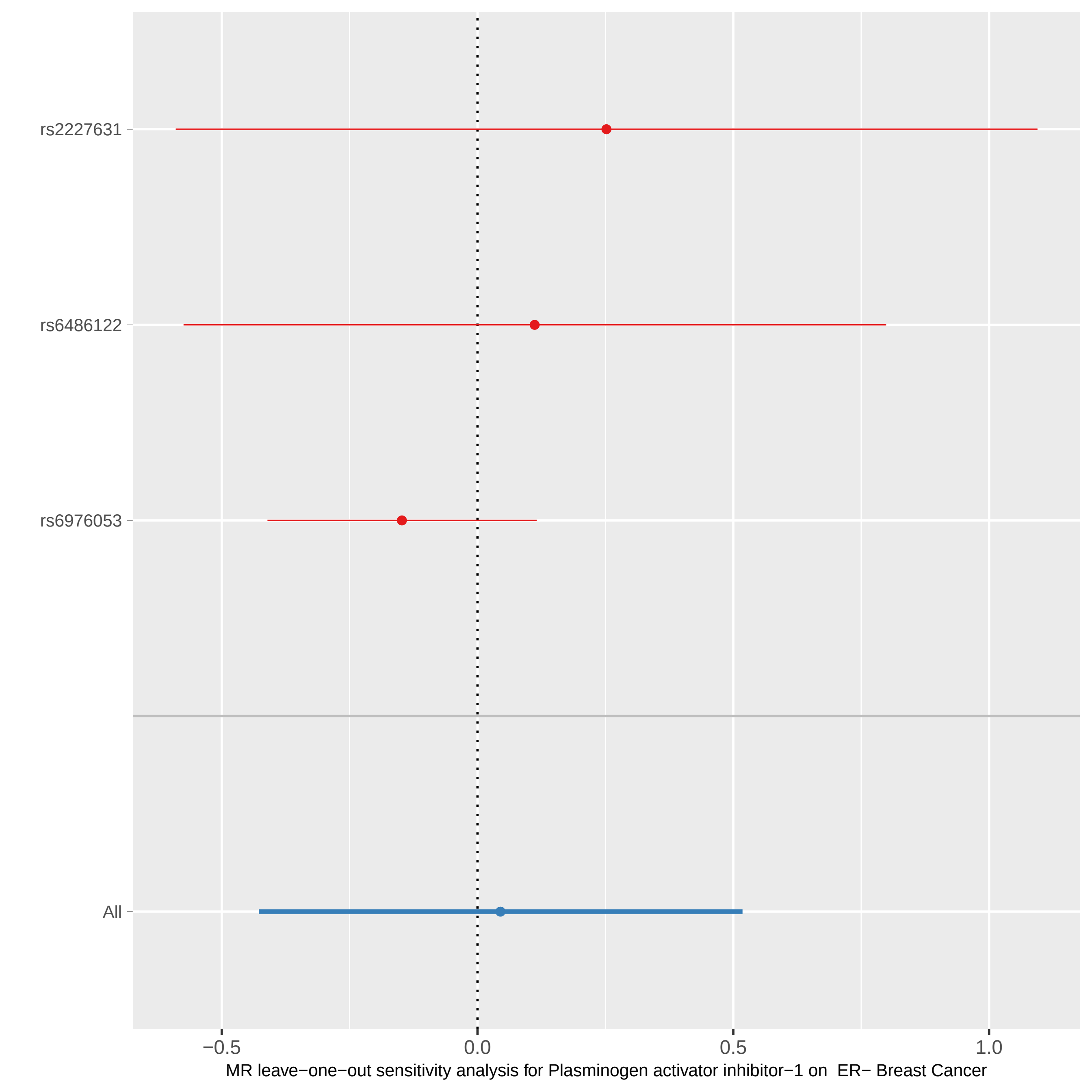
rs2227631
rs6486122
rs6976053
All
−0.5
0.0
0.5
1.0
MR leave−one−out sensitivity analysis for Plasminogen activator inhibitor−1 on ER− Breast Cancer

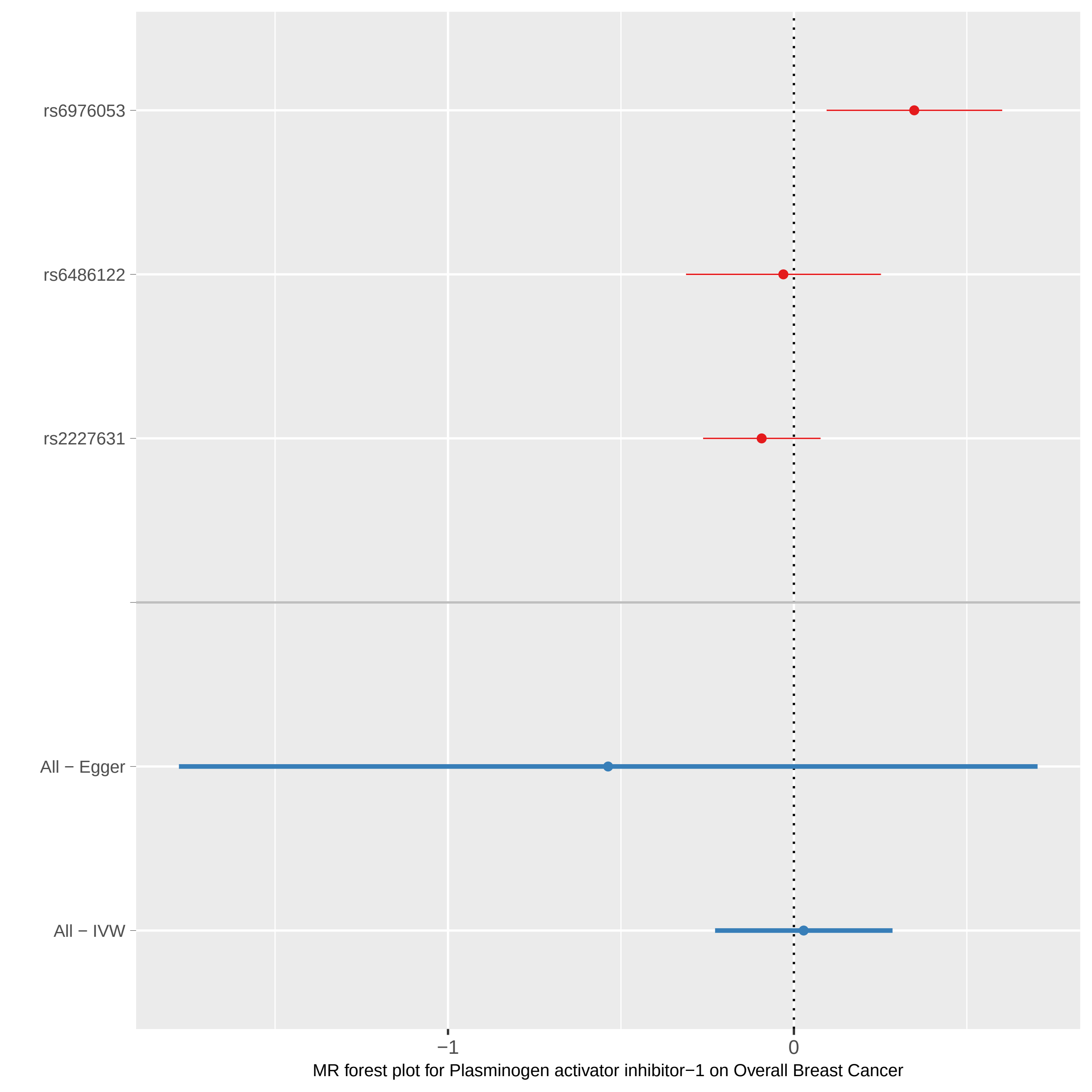
rs6976053
rs6486122
rs2227631
All − Egger
All − IVW
−1
0
MR forest plot for Plasminogen activator inhibitor−1 on Overall Breast Cancer

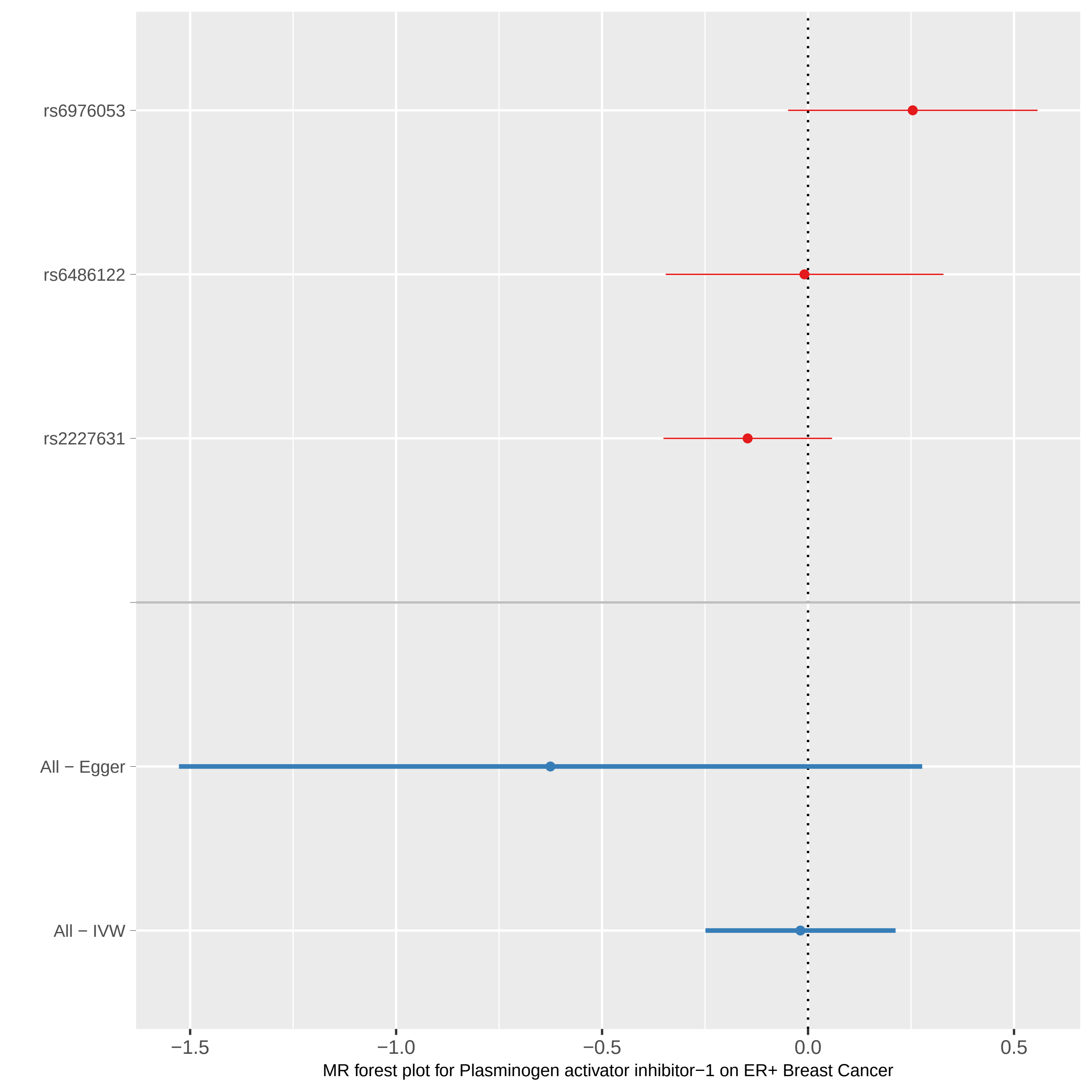
rs6976053
rs6486122
rs2227631
All − Egger
All − IVW
−1.5
−1.0
−0.5
0.0
0.5
MR forest plot for Plasminogen activator inhibitor−1 on ER+ Breast Cancer

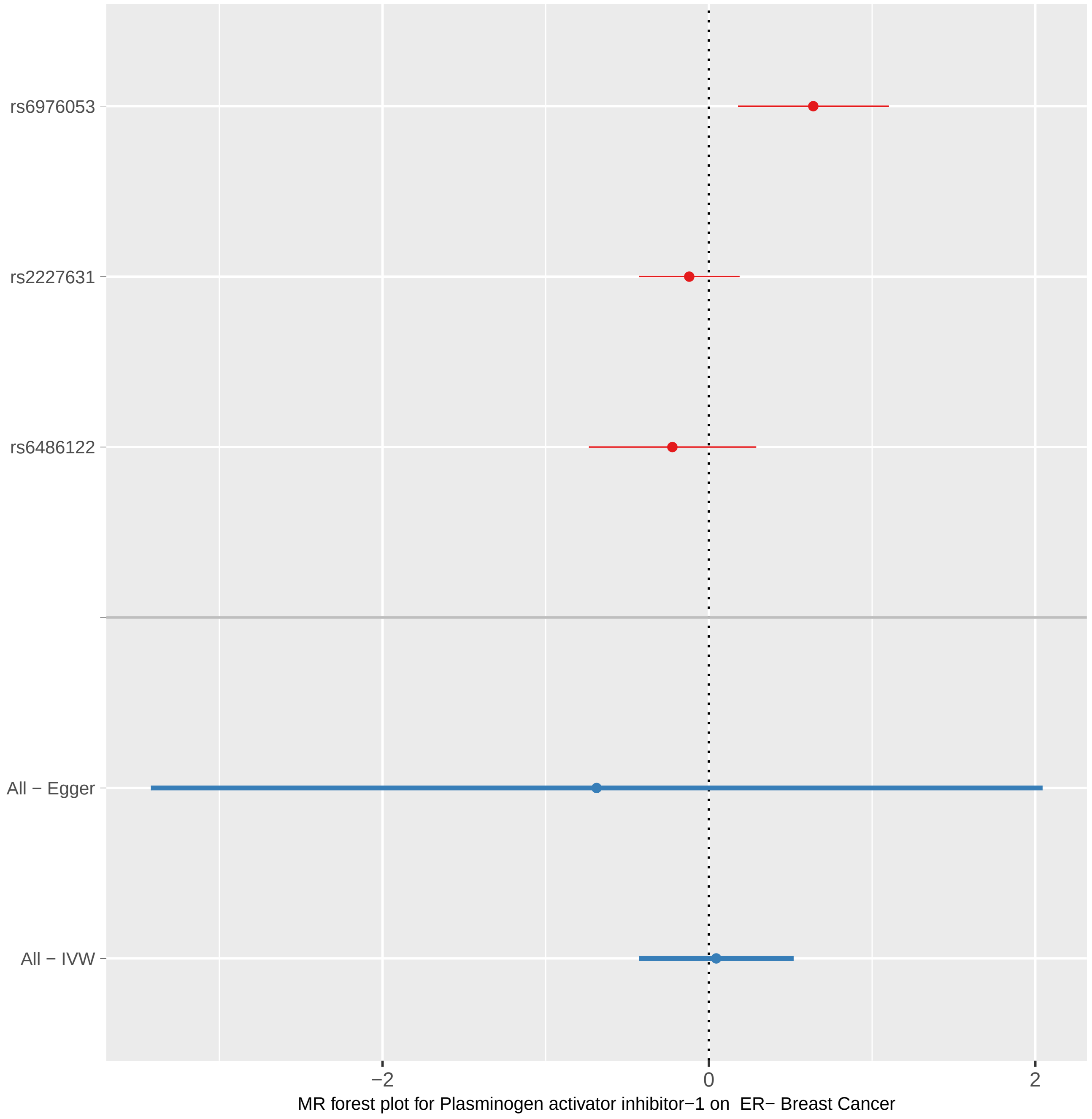
rs6976053
rs2227631
rs6486122
All − Egger
All − IVW
−2
0
2
MR forest plot for Plasminogen activator inhibitor−1 on ER− Breast Cancer

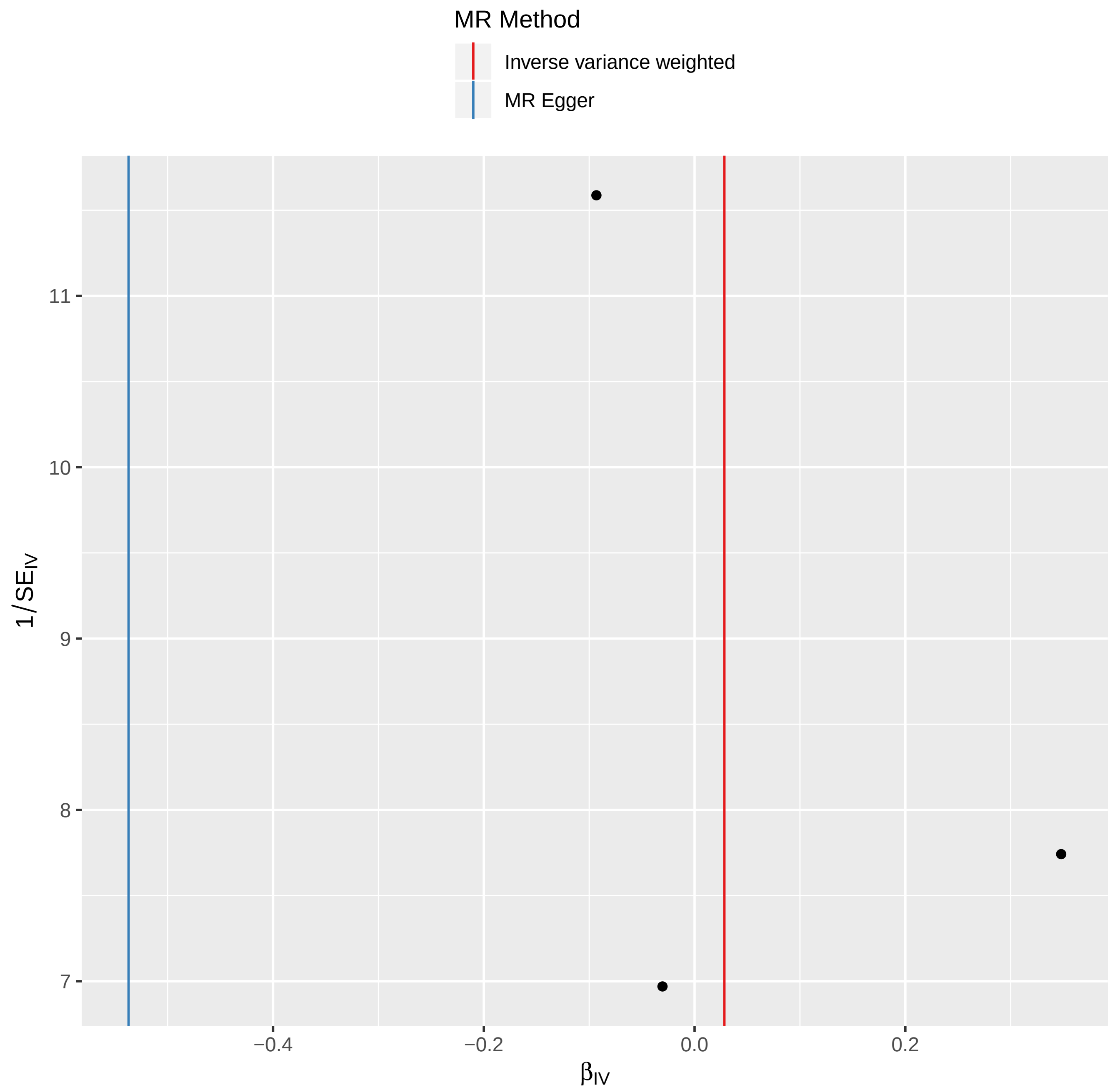
MR Method
Inverse variance weighted
MR Egger
1/SE_IV
β_IV
11
10
9
8
7
−0.4
−0.2
0.0
0.2

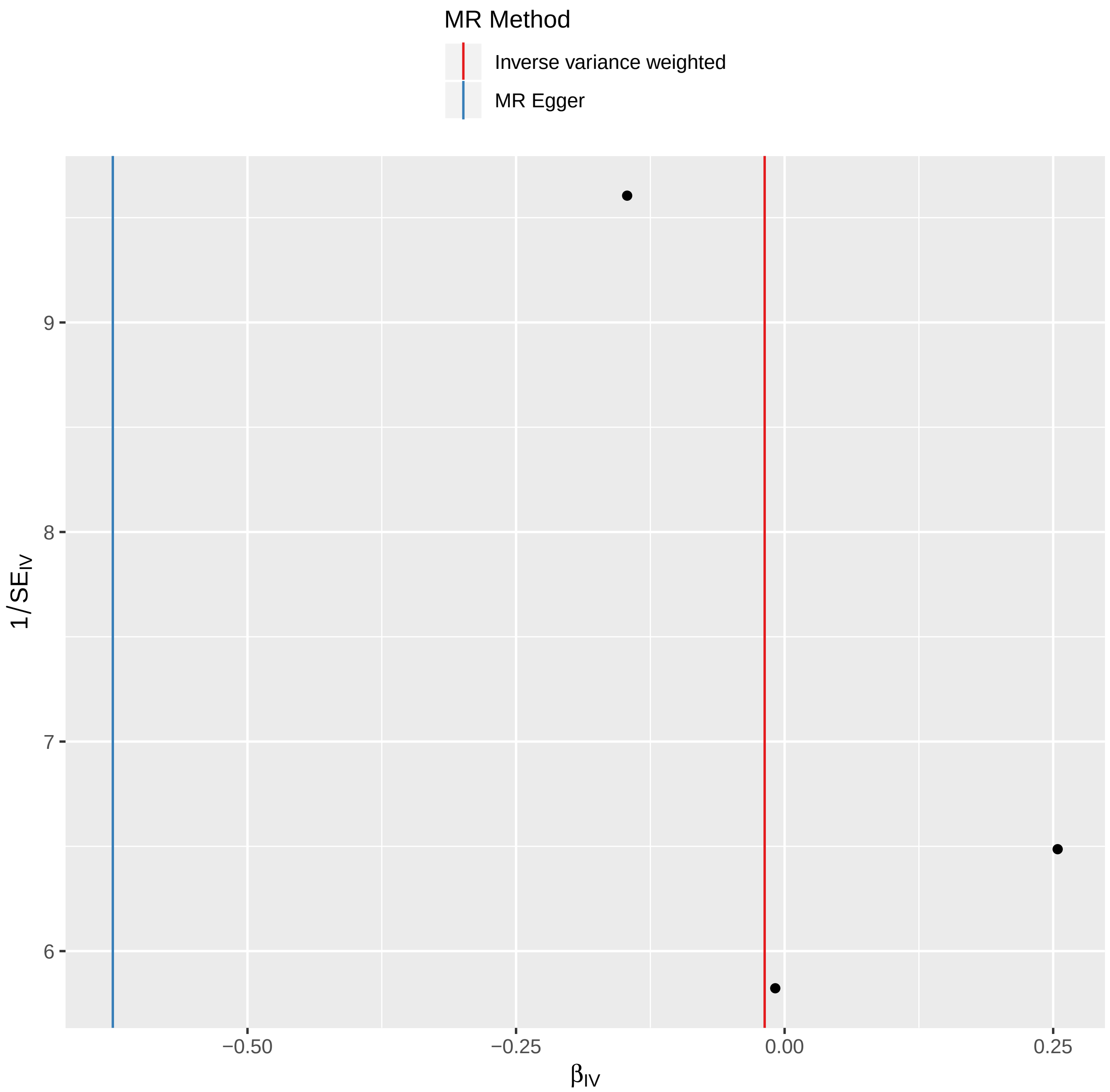
MR Method
Inverse variance weighted
MR Egger
9
8
7
6
1/SE_IV
−0.50
−0.25
0.00
0.25
β_IV

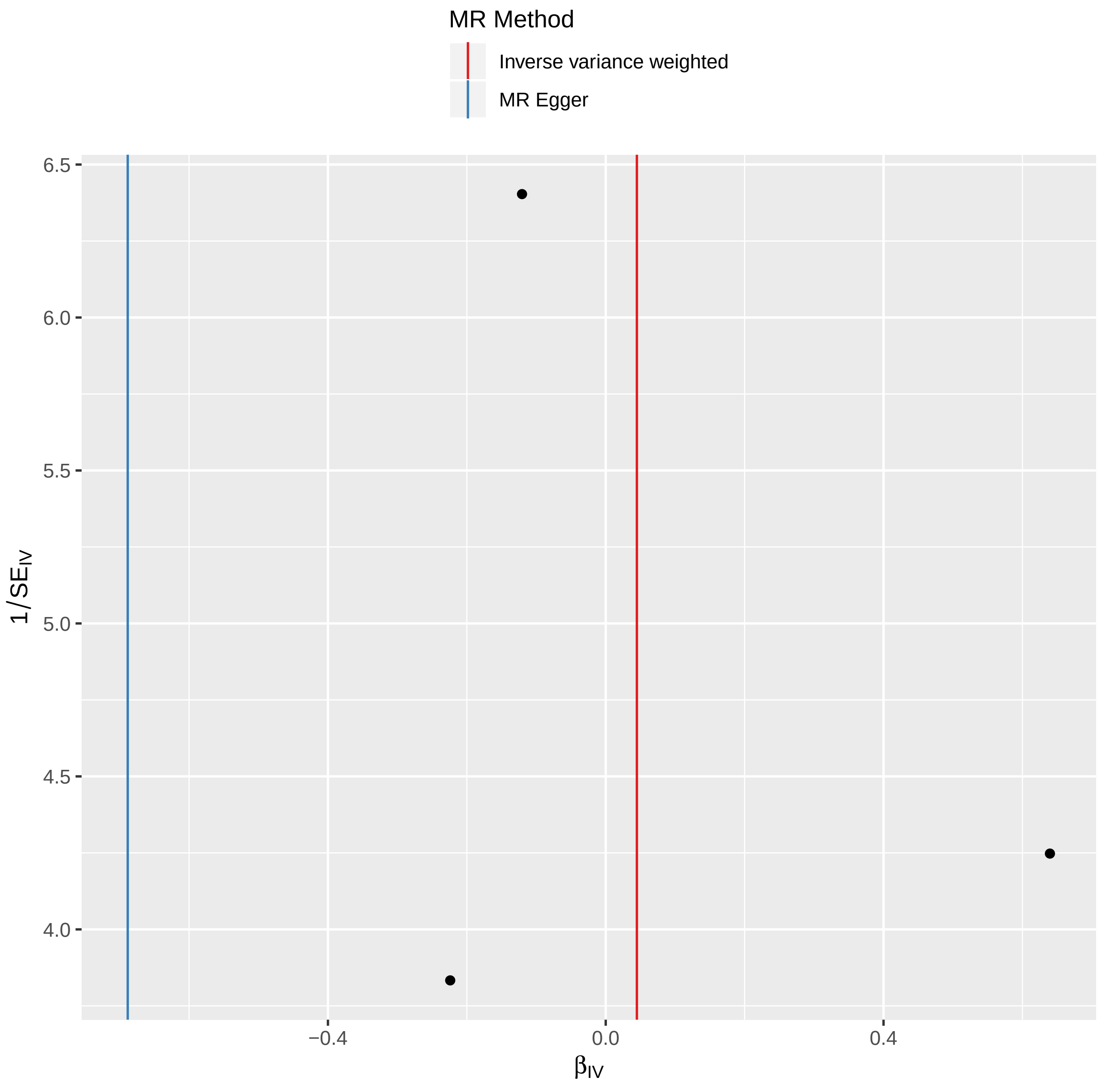
MR Method
Inverse variance weighted
MR Egger
6.5
6.0
5.5
5.0
4.5
4.0
$1/SE_{IV}$
−0.4
0.0
0.4
β_{IV}
